# Integrated computational screening and biochemical validation identify natural small molecules targeting Aβ aggregation and dissociation

**DOI:** 10.3389/jpps.2026.17216

**Published:** 2026-10-20

**Authors:** Hyewon Lee, Vijay Kumar, Soo Jung Shin, Yunkwon Nam, Sinae Jang, Hyeon soo Kim, Minseok Lee, Gyu Yong Song, Eun-Kyung Lim, Han-Jung Chae, Minho Moon

**Affiliations:** 1 School of Pharmacy, Jeonbuk National University, Jeonju, Republic of Korea; 2 Department of Biochemistry, College of Medicine, Konyang University, Daejeon, Republic of Korea; 3 Research Institute for Dementia Science, Konyang University, Daejeon, Republic of Korea; 4 College of Pharmacy, Chungnam National University, Daejeon, Republic of Korea; 5 AREZ Co. Ltd., Daejeon, Republic of Korea; 6 Bionanotechnology Research Center, Korea Research Institute of Bioscience and Biotechnology (KRIBB), Daejeon, Republic of Korea; 7 Department of Nanobiotechnology, KRIBB School of Biotechnology, University of Science and Technology, Daejeon, Republic of Korea; 8 School of Pharmacy, Sungkyunkwan University, Suwon, Republic of Korea; 9 Non-Clinical Evaluation Center Biomedical Research Institute, Jeonbuk National University Hospital, Jeonju, Republic of Korea

**Keywords:** Alzheimer’s disease, amyloid-β, Aβ aggregation and dissociation, computational screening, thioflavin T assay

## Abstract

Alzheimer’s disease (AD) is characterized by the accumulation of amyloid-β (Aβ), a pathological hallmark implicated in disease onset and progression. Aβ undergoes self-assembly from monomers into soluble oligomers, protofibrils, insoluble fibrils, and extracellular plaques. Our previous studies have shown that Jowiseungchungtang, which consists of 14 medicinal herbs, inhibits Aβ aggregation and ameliorates Aβ-mediated pathology in AD models. However, the active small molecules underlying these effects remain unidentified. To identify small molecules from these 14 medicinal herbs, we constructed a library of 1,751 compounds and applied an integrated *in silico* and *in vitro* workflow. First, these 1,751 compounds were filtered based on drug-likeness and predicted blood-brain barrier permeability, yielding 566 candidates. Second, molecular docking of 566 selected compounds against Aβ conformers identified five candidates predicted to interact with aggregation-prone residues critical for Aβ aggregation and fibril stabilization. Third, Thioflavin T assays demonstrated that four compounds—1-monopalmitin, isobutyl octyl oxalate, pentadecanoic acid, and dimethyl azelate—significantly inhibited Aβ_42_ aggregation. Additionally, isobutyl octyl oxalate and pentadecanoic acid disaggregated preformed Aβ_42_ aggregates. Fourth, molecular dynamics simulations were performed to examine the short-timescale behavior of the candidate compounds with the Aβ_17–42_ dimer, showing transient ligand–Aβ interactions associated with structural rearrangements at the dimer interface. Pharmacokinetic predictions supported drug-likeness, showing blood-brain barrier permeability and systemic clearance. Collectively, these findings identify four Jowiseungchungtang (JWS)-derived candidate compounds with Aβ aggregation-inhibitory activity and provide a mechanistic and pharmacokinetic basis for further development of these compounds as potential therapeutic agents in AD.

## Introduction

Alzheimer’s disease (AD) represents the predominant cause of dementia and manifests as progressive cognitive impairment with memory loss. Among the pathological features of AD, amyloid-β (Aβ) accumulation has been widely regarded as a central initiating cause of disease pathogenesis [1, 2]. According to the amyloid cascade hypothesis, dysregulation of Aβ production and clearance initiates a pathological process that ultimately leads to synaptic failure, neuronal degeneration, and cognitive decline [3–5]. Aβ monomers progressively assemble into soluble oligomers and protofibrils, as well as insoluble fibrils, ultimately giving rise to extracellular amyloid plaques, one of the major pathological hallmarks of AD [2, 6, 7]. Among these species, soluble oligomers and protofibrils of Aβ are considered particularly neurotoxic and are thought to play a major role in synaptic dysfunction and neuronal death [8, 9]. Notably, soluble and oligomeric forms of Aβ accumulate in both intracellular and extracellular compartments, with intraneuronal Aβ accumulation implicated in early synaptic dysfunction, neuronal degeneration, and progression of AD [1, 10, 11]. Consistent with its critical role in AD pathogenesis, Aβ has been designated as a core biomarker in the National Institute on Aging-Alzheimer’s Association (NIA-AA) research framework and is widely used in the diagnosis of AD [12–14]. Moreover, Aβ represents a major therapeutic target in AD, as supported by clinical trials of anti-amyloid monoclonal antibodies [15], such as lecanemab and donanemab, which effectively clear Aβ plaques and slow cognitive decline in patients with early AD [16, 17]. However, Aβ-targeting antibody-based therapies are associated with important limitations, including amyloid-related imaging abnormalities (ARIA), the need for repeated intravenous administration and magnetic resonance imaging (MRI) monitoring, and modest clinical benefits given the treatment burden and high cost [18–20]. Collectively, these limitations highlight the need for alternative Aβ-targeting approaches, particularly small-molecule candidates [21]. In this regard, critical sequence regions within Aβ drive its aggregation by promoting hydrophobic packing and β-sheet stabilization [22]. In particular, hydrophobic regions within Aβ, including ^17^LVF^19^, ^32^IGL^34^, and ^41^IA^42^, facilitate early self-assembly, whereas the central ^17^LVFFAED^23^ and C-terminal ^30^AIIGLMV^36^ segments promote β-sheet formation and fibril stabilization [23], supporting a stepwise conformational change of Aβ from disordered form to β-sheet-rich oligomers, protofibrils, and fibrils. Accordingly, strategies that simultaneously block key hydrophobic interfaces and disrupt β-sheet stabilization may represent a rational approach to effectively inhibit the self-assembly and structural maturation of Aβ [23–26].

Natural small molecules have emerged as attractive candidates for central nervous system (CNS) drug discovery, owing to their exceptional structural diversity, suitable physicochemical properties, potential to penetrate the blood-brain barrier (BBB), and a lower propensity for adverse effects, thereby facilitating effective brain delivery and target engagement [27, 28]. Importantly, Aβ aggregation is driven by hydrophobic interactions and β-sheet stabilization. Small molecules capable of binding to these aggregation-prone regions may interfere with both Aβ self-assembly and the subsequent structural maturation into stable fibrils. In this regard, traditional oriental medicines, which contain diverse natural small molecules, can provide a valuable source for the identification of inhibitors of Aβ aggregation. Jowiseungchungtang (JWS), comprising 14 medicinal herbs, has long been prescribed for cognitive impairment and dementia-related symptoms [29]. Prospective clinical studies indicate that long-term administration of JWS may delay cognitive decline and disease progression in patients with early-stage AD [29–31]. Notably, our previous study demonstrated two key effects: 1) JWS directly inhibited Aβ_42_ aggregation in thioflavin T (ThT) assays, and 2) oral administration reduced Aβ plaque deposition in the cerebral cortex of 5XFAD mice [32]. Importantly, our study reported that JWS exhibited no significant effects on tau aggregation or dissociation *in vitro* [33], suggesting specificity of JWS toward Aβ pathology. These findings suggest that the 14 medicinal herbs contain bioactive small molecules capable of modulating Aβ pathology. However, despite supporting evidence of the modulatory effects of JWS on Aβ pathology, the specific bioactive small molecules responsible for this activity remain unidentified. Therefore, it is necessary to identify the active small molecules from the 14 medicinal herbs that are most strongly associated with modulating Aβ aggregation.

To address this limitation, the present study employed an integrated *in silico* and *in vitro* experimental approach to evaluate JWS-derived compounds with inhibitory potential against Aβ aggregation (Figure 1). The novelty of this approach lies not in the general concept of natural products as Aβ aggregation inhibitors, but in the sequential integration of a large JWS-derived compound library with CNS-oriented filtering, multi-conformational Aβ docking, and biochemical validation of both aggregation inhibition and dissociation of preformed aggregates. This strategy enabled the identification of previously underexplored JWS-derived compounds with activity against different stages of Aβ aggregation. We first assembled a library of 2,253 compounds derived from the 14 medicinal herbs constituting JWS and retained 1,751 unique compounds after removing duplicates. These 1,751 compounds were then screened for drug-likeness and BBB permeability, yielding 566 candidate molecules qualified for both conditions. Next, molecular docking simulation was performed to assess the predicted binding interactions of the 566 candidate compounds with multiple Aβ conformers, leading to the selection of five JWS-derived compounds. Additionally, to validate the computational predictions, ThT assays were conducted to evaluate the effects of the five selected compounds on Aβ_42_ aggregation and dissociation *in vitro*. Four of the five selected compounds exhibited significant inhibition of Aβ_42_ aggregation in ThT assays, whereas two compounds effectively dissociated preformed Aβ_42_ aggregates. Based on the ThT assay results, molecular dynamics (MD) simulations were performed to examine the short-timescale behavior of the four compounds with the Aβ_17–42_ dimer. Finally, pharmacokinetic properties and BBB permeability of the four compounds were predicted to assess their drug-likeness. This study was conducted with two main objectives: 1) to construct a library of JWS-derived compounds to identify inhibitors of Aβ aggregation and inducers of disaggregation, and 2) to prioritize candidate compounds for further investigation of their potential contribution to the anti-Aβ activity of JWS. Collectively, our integrated screening and validation approach identified four JWS-derived candidate compounds—1-monopalmitin, isobutyl octyl oxalate, pentadecanoic acid, and dimethyl azelate—with anti-aggregation activity against Aβ. Additionally, isobutyl octyl oxalate and pentadecanoic acid exhibited disaggregative activity on preformed Aβ aggregates, providing a basis for the further development of small-molecule therapeutics for AD.

**FIGURE 1:**
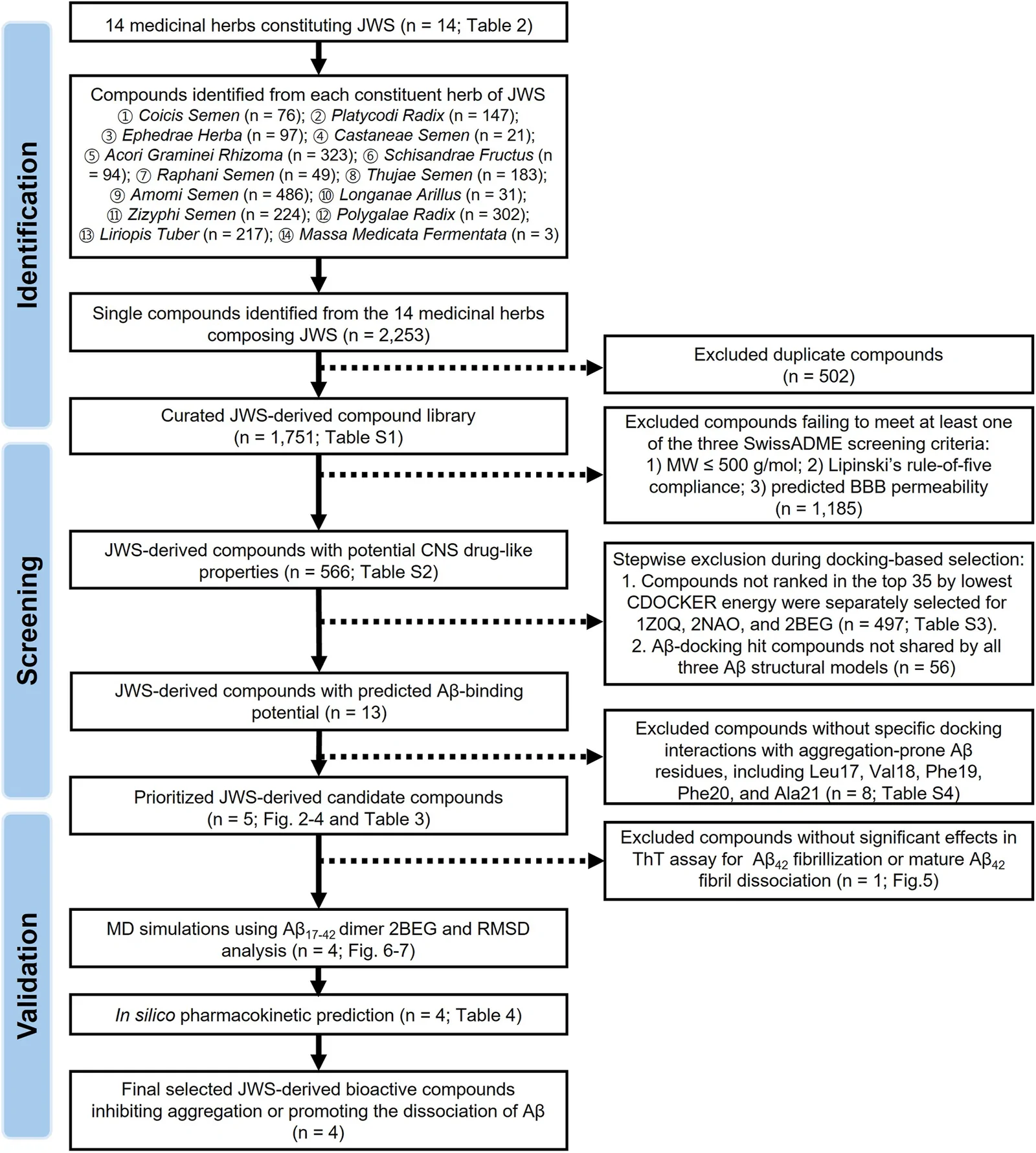
Flowchart of the selection process for JWS-derived compounds.

## Materials and methods

### Preparation of the JWS compound library

The compound library for the integrated *in silico* and *in vitro* workflow was constructed from the 14 medicinal herbs constituting JWS (Figure 1). These herbs include *Acori Graminei Rhizoma, Amomi Semen, Castaneae Semen, Coicis Semen, Ephedrae Herba, Liriopis Tuber, Longanae Arillus, Massa Medicata Fermentata, Platycodi Radix, Polygalae Radix, Raphani Semen, Schisandrae Fructus, Thujae Semen,* and *Zizyphi Semen*. Subsequently, small molecules from the constituents of each herb were systematically searched in public databases, including PubMed and TM-MC 2.0^1^ [34]. This search process yielded 2,253 single compounds. After removing duplicate entries, the final library comprised 1,751 unique compounds. The Simplified Molecular Input Line Entry System (SMILES) strings for these compounds were obtained from PubChem^2^ and TM-MC 2.0 to use as input for subsequent computational analyses.

### *In silico* screening of JWS-derived compounds for brain-targeting properties

An initial *in silico* screening step was performed to select JWS-derived compounds with drug-like properties and predicted BBB permeability. The web-based tool SwissADME^3^ was used to evaluate the 1,751 compounds curated from the JWS-derived compound library using a combined filtering approach. Two criteria were applied simultaneously: 1) compliance with Lipinski’s rule of five to assess basic oral drug-likeness and 2) predicted BBB permeability based on the BOILED-Egg model to assess CNS-targeting suitability. Compounds were selected only when they met both criteria. This filtering process excluded 1,185 compounds, leading to the selection of 566 JWS-derived compounds with potential brain-targeting properties.

### Molecular docking simulation

First, JWS-derived 566 compounds were prepared as ligands in BIOVIA Discovery Studio Client 2025 (Dassault Systèmes, San Diego, CA, USA) according to the following protocol described in our previous study [35]. Ligand structures were imported, and ionization was performed based on pH conditions, with automatic protonation of acidic groups and deprotonation of basic groups. Tautomeric forms were generated by exhaustive enumeration up to a maximum of 10 variants, excluding carbon atoms as hydrogen donors, maintaining aromaticity patterns, and limiting tautomerization to diamide moieties. Stereoisomers were generated for unspecified stereocenters and stereobonds, excluding bridgehead atoms, retaining marked stereochemistry, and correcting valence errors. Three-dimensional (3D) coordinates were assigned, and hydrogen atoms were optimized for proper spatial orientation. As the Aβ aggregation process involves various structural forms, it is crucial to use representative structures as targets [35]. Three major structures of Aβ–1Z0Q, 2NAO, and 2BEG–were obtained from the protein data bank (PDB) for use in molecular docking and dynamics simulations. Each structure was optimized using standard protein preparation protocols: imported protein molecules underwent loop building based on SEQRES definitions for loops up to 20 residues in length, without using Looper. The chemistry at HARvard Macromolecular Mechanics (CHARMm) minimization was applied with flexible stem residues set to 0. Protonation was performed using a protein dielectric constant of 10, pH of 7.4, ionic strength of 0.145, and an energy cutoff of 0.9. In advanced settings, the CHARMm force field was used, ligands were retained, waters were excluded, and disulfide bridges were handled with the default settings. Detailed information on the Aβ structures used in the simulations is summarized in Table 1. An α-helical monomeric structure of Aβ, PDB ID: 1Z0Q, was selected to represent the monomeric state. In contrast, a β-sheet monomeric conformation, PDB ID: 2NAO, was chosen to model a state with high aggregation propensity. Additionally, the pentameric forms of Aβ derived from PDB ID: 2BEG, representing the neurotoxic oligomeric states, were included as targets for molecular docking studies. By targeting multiple structural states, we aimed to increase the probability of discovering inhibitors that can act at various stages of the aggregation process.

**TABLE 1:** Structural models of Aβ used for molecular docking and molecular dynamics simulations.

PDB IDs	Original PDB structure	Structure used in simulation	Sequence	Application
1Z0Q	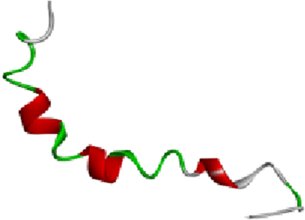	α-helical monomer 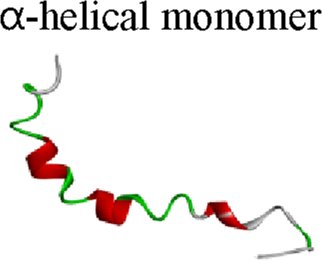	^1^DAEFRHDSGYEVHHQKLVFFAEDVGSNKGAIIGLMVGGVVIA^42^	Molecular docking simulation
2NAO	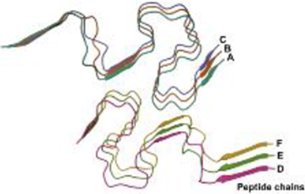	β-sheet monomer 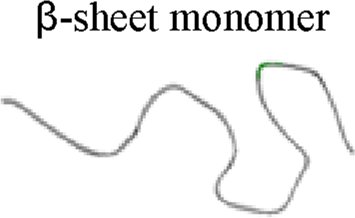	^1^DAEFRHDSGYEVHHQKLVFFAEDVGSNKGAIIGLMVGGVVIA^42^	
2BEG	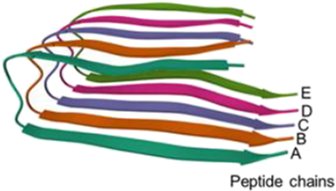	Pentameric form of 2BEG 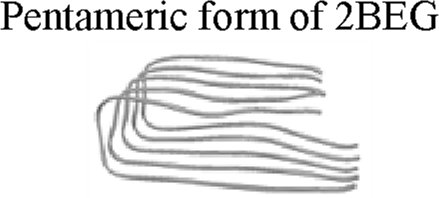	^17^LVFFAEDVGSNKGAIIGLMVGGVVIA^42^	
		Dimeric form of 2BEG 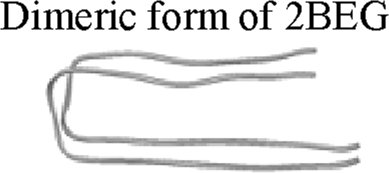	^17^LVFFAEDVGSNKGAIIGLMVGGVVIA^42^	Molecular dynamics simulation

The α-helical monomer PDB, 1Z0Q, β-sheet monomer PDB, 2NAO, and pentameric oligomer PDB, 2BEG, were used for molecular docking simulations. The dimeric form of 2BEG, was used for molecular dynamics simulations. Aβ structures are shown in ribbon representation. The α-helical monomer is colored according to secondary structure, with α-helical regions shown in red and non-helical regions shown in green and gray. Individual chains in the pentameric oligomer are labeled A–E in different colors.

Molecular docking simulations were performed using the CDOCKER algorithm within the Discovery Studio 2025 software. CDOCKER is a CHARMm-based algorithm that employs a semi-flexible docking approach, which accounts for the conformational flexibility of the ligand. Key simulation parameters included generating 10 top docking poses per compound and a pose cluster radius of 0.5 Å. The docking was performed using 10 random conformations, 1,000 dynamics steps at a target temperature of 1,000 Kelvin (K) and included electrostatic interactions. Simulated annealing was performed using 2,000 heating steps to 700 K followed by 5,000 cooling steps to 300 K. Semi-flexible ligand docking of the selected small molecules was carried out with the CHARMm force field. These docking settings produced a range of energy scores, including CDOCKER Interaction Energy. The following energy values were used to evaluate and rank the docking performance of the candidate compounds: CDOCKER Energy, CDOCKER Interaction Energy and Ligand Strain Energy. The Ligand Strain Energy represents the internal energy change associated with ligand deformation upon binding and was calculated by subtracting the −CDOCKER Energy from the −CDOCKER Interaction Energy and taking the absolute value. The 2D-ligand interactions were visualized using the visualization module in Discovery Studio 2025.

### Materials

Thioflavin T and synthetic Aβ_42_ peptide were purchased from Tokyo Chemical Industry Co., Ltd. (Tokyo, Japan) and Bachem AG (Bubendorf, Switzerland), respectively. Morin was obtained from Sigma-Aldrich (St. Louis, MO, USA). Five compounds were purchased: 1-monopalmitin was obtained from Chemscene (Monmouth Junction, NJ, USA); dibutyl sebacate and pentadecanoic acid were purchased from Combi-Blocks (San Diego, CA, USA); dimethyl azelate and isobutyl octyl oxalate were procured from An-gene (Shanghai, China).

### ThT assay for Aβ_42_ aggregation and dissociation

The inhibitory effects of the five compounds—1-monopalmitin, isobutyl octyl oxalate, pentadecanoic acid, dimethyl azelate, and dibutyl sebacate—on Aβ_42_ aggregation were assessed using a ThT fluorescence assay. Synthetic Aβ_42_ peptides were initially dissolved in 0.1 M aqueous ammonia. For the assay, this peptide stock solution was adjusted to 25 µM using triple-distilled water and transferred into black 96-well plates. Compound stock solutions were formulated using dimethyl sulfoxide (DMSO) as the solvent. Each well was treated with the five compounds at final concentrations of 2.5, 25, and 250 μM. Morin at 100 µM and distilled water were used as the positive control and vehicle, respectively. Incubation of the ThT-assay mixtures was carried out at 37 °C for 48 h in a 50 mM glycine buffer at pH 8.9 containing 15 μM ThT. To evaluate the potential effect of DMSO on Aβ_42_ aggregation, Aβ_42_ was treated with DMSO at final concentrations corresponding to those present in each compound-treated group and compared with the distilled water-treated control. This experiment was performed in technical duplicate within a single experiment (Supplementary Figure S2).

To assess the dissociation effects, Aβ_42_ fibrils were first prepared by incubating 25 μM Aβ_42_ peptide in triple-distilled water at 37 °C for 24 h in black 96-well plates. The preformed Aβ_42_ fibrils were then treated with each compound at final concentrations of 2.5, 25, and 250 μM at 37 °C for 48 h in black 96-well plates. Distilled water and crude JWS were used as the vehicle and positive control, respectively. Fluorescence intensity was recorded on a SpectraMax iD3 Multi-Mode Microplate Reader (Molecular Devices, San Jose, CA, USA), setting the excitation to 440 nm and emission to 484 nm. Both the aggregation and dissociation assays for the compound-treated conditions were performed in technical triplicate within a single experiment.

### Molecular dynamics simulations

MD simulations were performed using BIOVIA Discovery Studio Client 2025 with the CHARMm force field. We selected the best-ranked docking poses from the molecular docking and used them as starting structures for the MD simulations. A simulation of the ligand-free Aβ dimer was also performed for comparison. Prior to dynamics, the ligand-free Aβ_17–42_ dimer and each ligand-bound complex were solvated in Discovery Studio using the Explicit Periodic Boundary model with an orthorhombic cell and a minimum distance of 10 Å from the solute to the box boundary. Counterions were added, and Na^+^ and Cl^−^ were included at a salt concentration of 0.145 M. MD simulations were then conducted on the solvated systems using the standard dynamics cascade protocol. The simulation workflow consisted of four sequential steps: 1) minimization phase: initial geometry optimization *via* Steepest Descent algorithm for 100 iterations with an RMS gradient threshold of 1, followed by secondary minimization using the Adopted Basis Newton-Raphson method for 500 iterations at 0.1 RMS gradient, 2) heating phase: 50 picoseconds (ps) of simulation with a 2 femtosecond (fs) time step, initial temperature of 50 K ramped to a target of 300 K, velocity rescaling applied every 50 steps, and trajectory data recorded every 2 ps, 3) equilibration phase: 100 ps simulation at 300 K using 2 fs time step, with velocity adjustment every 50 steps, trajectory data recorded every 2 ps, and 4) production phase: 10,000 ps simulation with a 2 fs time step at a constant temperature of 300 K, carried out in NAMD. Production was run under isothermal–isobaric (NPT) ensemble conditions with a reference pressure of 1 atm, and trajectories were saved every 50 ps. Production MD runs were performed in triplicate for the ligand-free Aβ_17–42_ dimer and for each ligand-bound complex using independent initial velocities.

During minimization, equilibration, and NAMD production, the implicit solvent model was not used (Generalized Born λ = 0), because solvation had already been treated with explicit periodic water and 0.145 M NaCl. Nonbonded electrostatics used an internal dielectric constant of 1. The implicit-solvent dielectric constant of 80 shown in the Discovery Studio dynamics interface is a default field for continuum-solvent calculations and was not applied. Nonbond lists were generated with a 14 Å cutoff; electrostatic and van der Waals cutoffs were 12 Å and 10 Å, respectively. The Leapfrog Verlet algorithm was used for numerical integration of equations of motion. Total energy was analyzed over 1,000–10,000 ps after the initial relaxation phase. For each replicate, the mean energy over this window was calculated first; values are reported as the mean ± SD of the three replicate means. Total-energy profiles were used as descriptive indicators of energetic stationarity and replicate reproducibility within each simulated system. Because the ligand-free and ligand-bound systems differed in molecular composition and total atom number, their absolute mean total energies were not considered directly comparable across systems and were not used to rank ligand–Aβ interactions or to infer preservation of the Aβ_17–42_ dimer interface. These simulations were intended to assess short-timescale ligand behavior and early dimer-interface perturbation, not to compute equilibrium binding free energies.

### Trajectory analysis

The backbone root mean square deviation (RMSD) was used to quantify structural deviation of the Aβ_17–42_ dimer during production molecular dynamics. Analyses were performed in BIOVIA Discovery Studio Client 2025 with the Analyze Trajectory protocol. For each replica, RMSD was computed relative to the first frame of the production trajectory, which corresponded to the final snapshot of the preceding equilibration. Each frame was superimposed onto this reference by least-squares fitting of backbone atoms (N, Cα, C, and O) before the RMSD was evaluated on the same atom set. Water, counter-ions, hydrogen atoms, and ligand atoms were excluded from both the fit and the peptide RMSD. Three complementary RMSD series were calculated for every trajectory. Whole-dimer RMSD was obtained by fitting and scoring the backbone atoms of both peptide chains together. Chain-specific RMSD was obtained by defining separate atom groups for chain A and chain B and repeating the fit and scoring on each chain independently, so that intra-chain conformational change could be distinguished from relative motion of the two chains. RMSD values were analyzed in the 1,000–10,000 ps window after the initial relaxation phase. For each replica, the mean RMSD over this window was calculated first; values are reported as the mean ± standard deviation (SD) of the three independent replica means. These analyses were used to assess short-timescale structural persistence of the dimer and early ligand-dependent changes at the chain and interface levels. They were not used to compute equilibrium binding free energies or to infer long-timescale fibril stability.

### Prediction of pharmacokinetic properties

The drug-like properties of four JWS-derived compounds were assessed based on the absorption, distribution, metabolism, and excretion (ADME). The physicochemical characteristics of four natural small compounds were determined using the web-based program PreADMET^4^ (bioinformatics and molecular design research center, Incheon, Republic of Korea) and pkCSM-pharmacokinetics^5^ (Bio21 institute, the University of Melbourne, Australia) to predict the pharmacokinetic properties [36]. The PreADMET web-based software can predict several important properties of drugs, including logarithm of partition coefficient between n-octanol and water (LogP_o/w_), plasma protein binding (PPB), Caco-2 permeability, human intestinal absorption (HIA), BBB penetration, as well as CYP2C19 and CYP2D6 inhibition. Furthermore, the pkCSM-pharmacokinetic software can predict the total clearance of compounds.

### Statistical analysis

Statistical evaluations were carried out with GraphPad Prism 10.0 software. Group comparisons for the ThT assays were assessed via one-way analysis of variance (ANOVA) followed by Fisher’s LSD *post hoc* test. Statistical significance was defined as *p* < 0.05. Data from the ThT assays are presented as the mean ± standard error of the mean (SEM) of technical replicates within a single experiment. For the MD simulations, data from three independent simulation runs are presented as the mean ± standard deviation (SD), as specified in the corresponding figure legends.

## Results

### Construction and filtering of JWS-derived compound library

A comprehensive search was conducted across public databases, including PubMed and TM-MC 2.0, to identify all 14 herbs constituting the JWS formula (Table 2). This analysis initially yielded a total of 2,253 compounds, which were reduced to 1,751 unique structures after removal of duplicate entries (Supplementary Table S1). The initial filtering step involved evaluating drug-likeness based on Lipinski’s rule of five and predicting BBB permeability using the web-based tool SwissADME. Filtering using these two criteria identified 566 candidate compounds (Supplementary Table S2). The selection process prioritized compounds exhibiting favorable oral bioavailability, including high gastrointestinal absorption predicted by the BOILED-Egg model and BBB permeability, which are key pharmacokinetic properties for CNS-targeted therapeutics. Together, these early filters identified 566 JWS-derived compounds with favorable oral bioavailability and BBB permeability.

**TABLE 2:** The list of the 14 constituent herbs of JWS, including the specific amounts of each herb used in the formulation.

No.	Name	Weight (Gram)	Ratio (%)
1	*Coicis Semen*	8.0	12.50
2	*Platycodi Radix*	4.0	6.25
3	*Ephedrae Herba*	3.0	4.69
4	*Castaneae Semen*	8.0	12.50
5	*Acori Graminei Rhizoma*	4.0	6.25
6	*Schisandrae Fructus*	3.0	4.69
7	*Raphani Semen*	6.0	9.37
8	*Thujae Semen*	4.0	6.25
9	*Amomi Semen*	3.0	4.69
10	*Longanae Arillus*	6.0	9.37
11	*Zizyphi Semen*	4.0	6.25
12	*Polygalae Radix*	3.0	4.69
13	*Liriopis Tuber*	4.0	6.25
14	*Massa Medicata Fermentata*	4.0	6.25
​	Total	64.0	100

### Molecular docking simulation of JWS-derived compound library against various forms of Aβ

Effective Aβ-targeting therapeutics should both inhibit the formation of Aβ aggregates and promote the dissociation of pre-formed aggregates to slow AD progression [5, 23, 35]. To evaluate the binding of small molecules to Aβ, molecular docking was performed for 566 compounds against three Aβ conformations, including [1] the α-helical monomeric Aβ structure, PDB ID 1Z0Q [2], the β-sheet monomeric Aβ structure, PDB ID 2NAO, and [3] the pentameric Aβ structure, PDB ID 2BEG (Table 1). The three Aβ structures represent distinct conformational states associated with Aβ aggregation and fibril formation. Among the successfully docked compounds, docking against the α-helical monomeric Aβ structure yielded CDOCKER energy values ranging from 39.33 to −116.36 kcal/mol among 566 compounds. In addition, the docking against β-sheet monomeric Aβ structure yielded CDOCKER energy values ranging from 34.91 to −97.28 kcal/mol, whereas docking against pentameric Aβ structure yielded values ranging from 46.80 to −927.22 kcal/mol (Supplementary Table S3). The docking results were further analyzed according to the predefined selection criteria. First, the 566 compounds were ranked according to their CDOCKER energy for each Aβ structural model, and the top 35 compounds were selected using CDOCKER energy cutoff values of −21.89 kcal/mol for 1Z0Q, −18.72 kcal/mol for 2NAO, and −27.56 kcal/mol for 2BEG. Compounds consistently ranked within the top 35 across all three Aβ structural models were retained, resulting in 13 candidates (Supplementary Table S4). These 13 compounds were subsequently evaluated based on their docking interactions with key aggregation-prone residues within the central hydrophobic region of Aβ, specifically Leu17, Val18, Phe19, Phe20, and Ala21. Compounds showing direct interactions with one or more of these residues were prioritized, resulting in the selection of five compounds—1-monopalmitin, isobutyl octyl oxalate, pentadecanoic acid, dimethyl azelate, and dibutyl sebacate—for further experimental validation (Supplementary Figure S1).

To elucidate the specific binding modes, we explored the docking poses of these five compounds against all three Aβ conformations: the monomeric α-helical, the β-sheet–rich monomeric, and the pentameric forms. First, in the α-helical monomeric Aβ structure, 1-monopalmitin exhibited the lowest CDOCKER energy of −39.3 kcal/mol. It formed hydrogen bonds with Phe19 and Asp23, in addition to hydrophobic interactions that included π-alkyl contacts with His14 and Val18 (Table 3; Figure 2A). Isobutyl octyl oxalate displayed a CDOCKER energy of −24.6 kcal/mol and hydrogen bonding was observed with Phe19 and Asp23, accompanied by a hydrophobic alkyl interaction with Val18 and a hydrophobic π-alkyl interaction with His14 (Table 3; Figure 2B). Pentadecanoic acid showed a CDOCKER energy of −27.8 kcal/mol and established a hydrogen bond with His14, together with hydrophobic alkyl interactions involving Val18 (Table 3; Figure 2C). Dimethyl azelate had a CDOCKER energy of −25.1 kcal/mol and formed multiple hydrogen bonds with His14, Gln15, Phe19, Glu22, and Asp23, supplemented by hydrophobic alkyl interactions with Val18 (Table 3; Figure 2D). Dibutyl sebacate yielded a CDOCKER energy of −34.3 kcal/mol and interacted predominantly through hydrophobic alkyl interactions with Lys28 (Table 3; Figure 2E). In summary, 1-monopalmitin demonstrated the lowest CDOCKER energy toward the α-helical monomeric Aβ structure, whereas dimethyl azelate exhibited the greatest number of hydrogen interactions with multiple residues.

**TABLE 3:** CDOCKER energy values, ligand strain energies, and interaction residues of five JWS-derived compounds docked to three Aβ conformations.

Small molecules	Aβ structures	CDOCKER energy (kcal/mol)	Ligand strain energy (kcal/mol)	Hydrogen-bonding residues	Hydrophobic interaction residues	Other interaction residues
1-Monopalmitin	α-helical monomeric Aβ, PDB ID 1Z0Q	−39.3	0.9	Phe19, Asp23	π-Alkyl interaction with His14; Alkyl interaction with Val18	Van der Waals interactions with Asp7, Glu11, Gln15, Phe20, and Glu22
	β-sheet monomeric Aβ PDB ID 2NAO	−25.7	1.4	Lys16, Val18, Phe20	Alkyl interactions with Lys16, Val18, and Ala21	Van der Waals interactions with Leu17, His13, Gln15, and Phe19
	Pentameric Aβ PDB ID 2BEG	−39.4	3.4	-	Alkyl interactions with Leu17, Val40, and Ala42; π-Alkyl interaction with Phe19	Van der Waals interactions with Val18, Leu17, Phe19, Gly37, Gly38, Val39, Val40, and Ala42
Isobutyl octyl oxalate	α-helical monomeric Aβ, PDB ID 1Z0Q	−24.6	0.1	Phe19, Asp23	Alkyl interactions with Val18; π-Alkyl interaction with His14	Van der Waals interactions with Glu11, Gln15, Glu22, and Asn27
	β-sheet monomeric Aβ PDB ID 2NAO	−22.7	0.6	Lys16, Val18, Phe20	Alkyl interaction with Ala21	Van der Waals interactions with Phe19, Glu22, and Ile32
	Pentameric Aβ PDB ID 2BEG	−35.6	5.6	Leu17	Alkyl interactions with Leu17 and Val40; π-Alkyl interaction with Phe19	Van der Waals interactions with Val18, Leu17, Gly37, Gly38, Val40, and Ala42
Pentadecanoic acid	α-helical monomeric Aβ, PDB ID 1Z0Q	−27.8	1.1	His14	Alkyl interaction with Val18	Van der Waals interactions with Phe19, Glu11, Glu22, Gln15, Asp23, and Asn27
	β-sheet monomeric Aβ PDB ID 2NAO	−30.6	0.5	-	Alkyl interactions with Lys16, Val18, Ala21, and Ile32	Van der Waals interactions with Leu17 and Glu22; Attractive charge interaction with Lys16
	Pentameric Aβ PDB ID 2BEG	−43.0	2.6	Leu17	Alkyl interactions with Leu17, Val40, and Ala42	Van der Waals interactions with Val18, Leu17, Phe19, Val40, and Ala42; Attractive charge interaction with Leu17
Dimethyl azelate	α-helical monomeric Aβ, PDB ID 1Z0Q	−25.1	2.8	His14, Gln15, Phe19, Glu22, Asp23	Alkyl interaction with Val18	Van der Waals interactions with Glu11 and Asn27
	β-sheet monomeric Aβ PDB ID 2NAO	−25.2	1.8	Lys16, Val18, Phe20	Alkyl interactions with Val18 and Ala21	Van der Waals interactions with Phe19 and Glu22
	Pentameric Aβ PDB ID 2BEG	−38.6	0.4	Leu17	Alkyl interactions with Phe19, Leu17, and Val40; π-Alkyl interaction with Phe19	Van der Waals interactions with Val18, Leu17, Phe19, Gly38, Val39, Val40, and Ala42
Dibutyl sebacate	α-helical monomeric Aβ, PDB ID 1Z0Q	−34.3	6.3	-	Alkyl interaction with Lys28	Van der Waals interactions with Glu11, His14, Gln15, Val18, Phe19, Glu22, Asp23, Asn27, and Ile31
	β-sheet monomeric Aβ PDB ID 2NAO	−33.9	7.5	Lys16, Val18, Ala21	Alkyl interactions with Ala21; π-Alkyl interaction with Phe19	Van der Waals interactions with Leu17, Phe20, and Ile32
	Pentameric Aβ PDB ID 2BEG	−47.6	2.4	-	Alkyl interactions with Phe19 and Val40; π-Alkyl interactions with Phe19 and Val40	Van der Waals interactions with Leu17, Val18, Phe19, Gly37, Gly38, Val39, Val40, and Ala42

**FIGURE 2:**
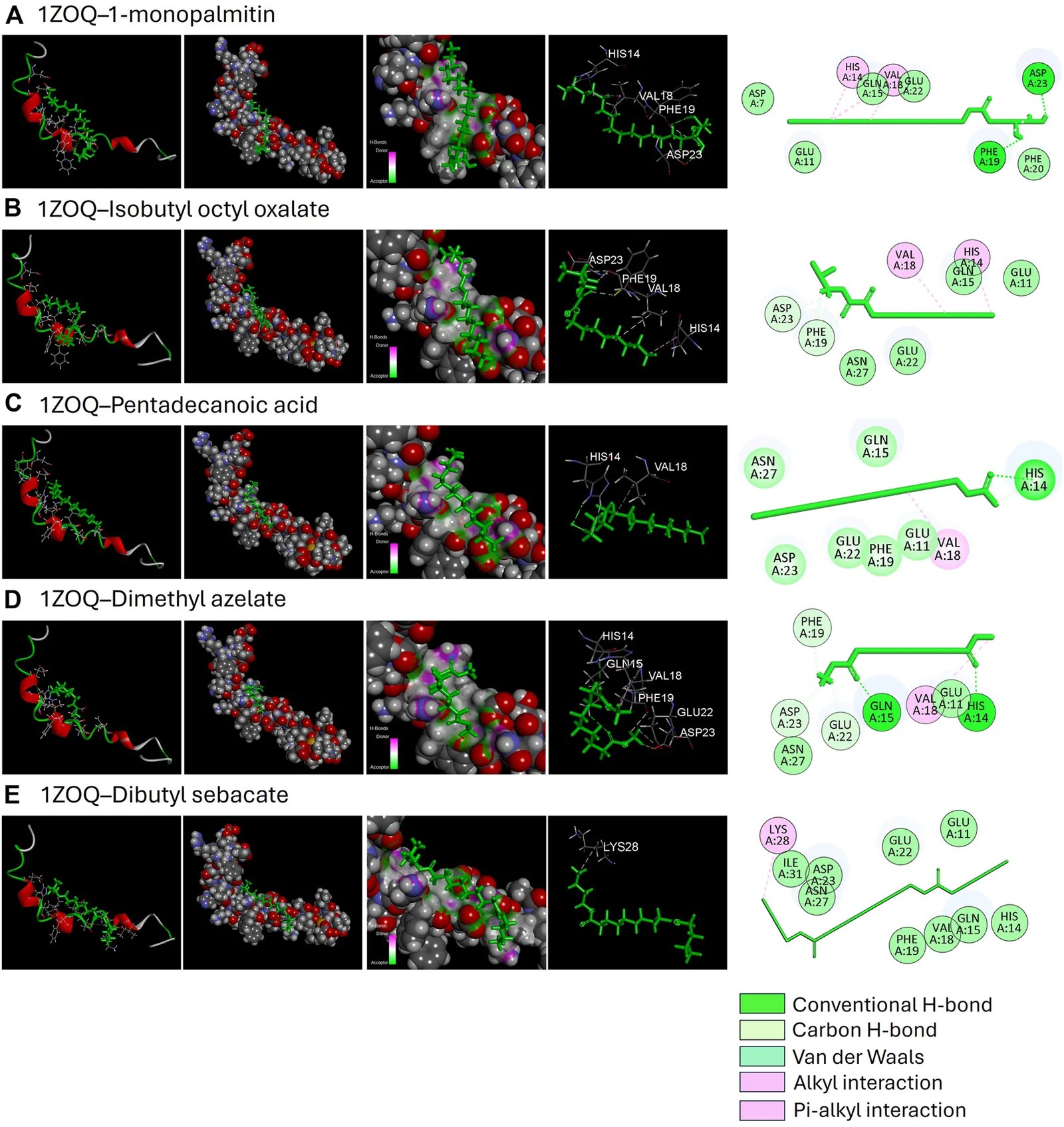
Molecular docking analysis of five JWS-derived compounds, including **(A)** 1-monopalmitin, **(B)** isobutyl octyl oxalate, **(C)** pentadecanoic acid, **(D)** dimethyl azelate, and **(E)** dibutyl sebacate, against the α-helical monomeric Aβ_42_ structure, PDB ID 1Z0Q. First panel shows the ligand positioned within the secondary structure of Aβ, with the α-helical structure of Aβ_42_ shown in red and green and interacting residues highlighted in gray. Second panel shows a detailed atomic representation of Aβ in a ball-and-stick model, with the ligand shown in green and carbon, oxygen, and nitrogen atoms shown as gray, red, and blue spheres, respectively. Third panel shows an enlarged view of the ligand-binding region within monomeric Aβ, with hydrogen-bond donor and acceptor surface indicated in purple and green, respectively. Fourth panel shows a simplified two-dimensional interaction diagram of ligand-binding residues in Aβ. The labels show the amino acid names in three-letter code and their positions in the Aβ peptide. The fifth panel shows a two-dimensional interaction map of ligand-binding residues in Aβ. Circles indicate interacting residues, and colors indicate interaction types defined in the bottom panel.

Second, in the β-sheet–rich monomeric Aβ structure, 1-monopalmitin showed a CDOCKER energy of −25.7 kcal/mol. It formed hydrogen bonds with Lys16, Val18, and Phe20, along with hydrophobic alkyl interactions involving Lys16, Val18, and Ala21 (Table 3; Figure 3A). Similarly, isobutyl octyl oxalate exhibited a CDOCKER energy of −22.7 kcal/mol and formed hydrogen bonds with Lys16, Val18, and Phe20, together with hydrophobic interactions involving Ala21 (Table 3; Figure 3B). Pentadecanoic acid displayed the most favorable CDOCKER energy of −30.6 kcal/mol and formed hydrophobic alkyl interactions with Lys16, Val18, Ala21, and Ile32 (Table 3; Figure 3C). Dimethyl azelate had a CDOCKER energy of −25.2 kcal/mol and formed hydrogen bonds with Lys16, Val18, and Phe20, in addition to hydrophobic interactions involving Val18 and Ala21 (Table 3; Figure 3D). Dibutyl sebacate showed a CDOCKER energy of −33.9 kcal/mol and formed hydrogen bonds with Lys16, Val18, and Ala21, along with hydrophobic alkyl interactions with Ala21 and hydrophobic π-alkyl interactions with Phe19 (Table 3; Figure 3E). Collectively, 1-monopalmitin, isobutyl octyl oxalate, and dimethyl azelate formed hydrogen bonds with the common residues Lys16, Val18, and Phe20, whereas hydrophobic interactions were predominantly observed with Ala21 across all five compounds.

**FIGURE 3:**
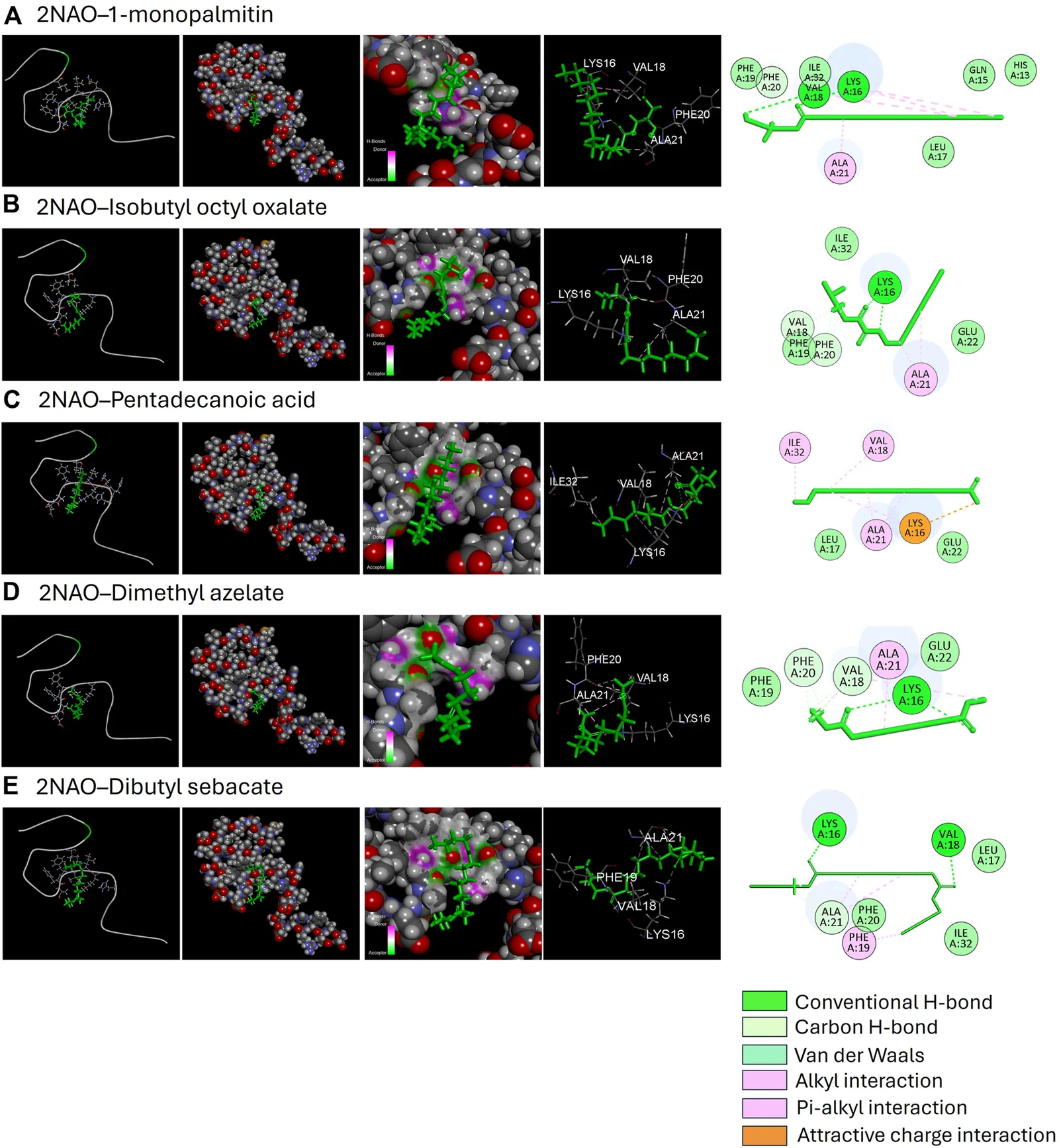
Molecular docking analysis of five JWS-derived compounds, including **(A)** 1-monopalmitin, **(B)** isobutyl octyl oxalate, **(C)** pentadecanoic acid, **(D)** dimethyl azelate, and **(E)** dibutyl sebacate, against the β-sheet–rich monomeric Aβ_42_ structure, PDB ID 2NAO. First panel presents the ligand positioned within the β-sheet structure of Aβ, with interacting residues highlighted in gray. Second panel presents an atomic-level ball-and-stick representation of Aβ showing ligand interactions, with the ligand shown in green and carbon, oxygen, and nitrogen atoms represented as gray, red, and blue spheres, respectively. Third panel presents an enlarged surface view of the ligand-binding region within monomeric Aβ, with hydrogen-bond donor and acceptor surfaces indicated in purple and green, respectively. Fourth panel presents a simplified two-dimensional diagram of ligand-binding residues in Aβ. The labels show the amino acid names in three-letter code and their positions in the Aβ peptide. Fifth panel presents a two-dimensional interaction map of ligand-binding residues in Aβ. Circles indicate interacting residues, and colors indicate interaction types defined in the bottom panel.

Third, in the pentameric Aβ structure, 1-monopalmitin showed a CDOCKER energy of −39.4 kcal/mol. 1-monopalmitin formed hydrophobic alkyl interactions with Leu17, Val40, and Ala42, as well as hydrophobic π-alkyl interactions with Phe19 (Table 3; Figure 4A). Isobutyl octyl oxalate exhibited a CDOCKER energy of −35.6 kcal/mol and formed a hydrogen bond with Leu17 and hydrophobic alkyl interactions with Leu17 and Val40, along with hydrophobic π-alkyl interactions with Phe19 (Table 3; Figure 4B). Pentadecanoic acid had a CDOCKER energy of −43.0 kcal/mol, forming a hydrogen bond with Leu17 and hydrophobic alkyl interactions with Leu17, Val40, and Ala42 (Table 3; Figure 4C). Dimethyl azelate showed a CDOCKER energy of −38.6 kcal/mol and formed a hydrogen bond with Leu17, along with hydrophobic alkyl interactions involving Phe19, Leu17, and Val40, and π-alkyl interactions with Phe19 (Table 3; Figure 4D). Lastly, dibutyl sebacate showed the most negative CDOCKER energy of −47.6 kcal/mol and predominantly formed both hydrophobic alkyl and π-alkyl interactions with Phe19 and Val40 (Table 3; Figure 4E). Collectively, hydrophobic interactions were predominantly observed with residues Leu17, Phe19, Val40, and Ala42. The pentameric form of Aβ exhibited fewer hydrogen bonds compared to the monomeric structures, whereas hydrophobic interactions became more prominent.

**FIGURE 4:**
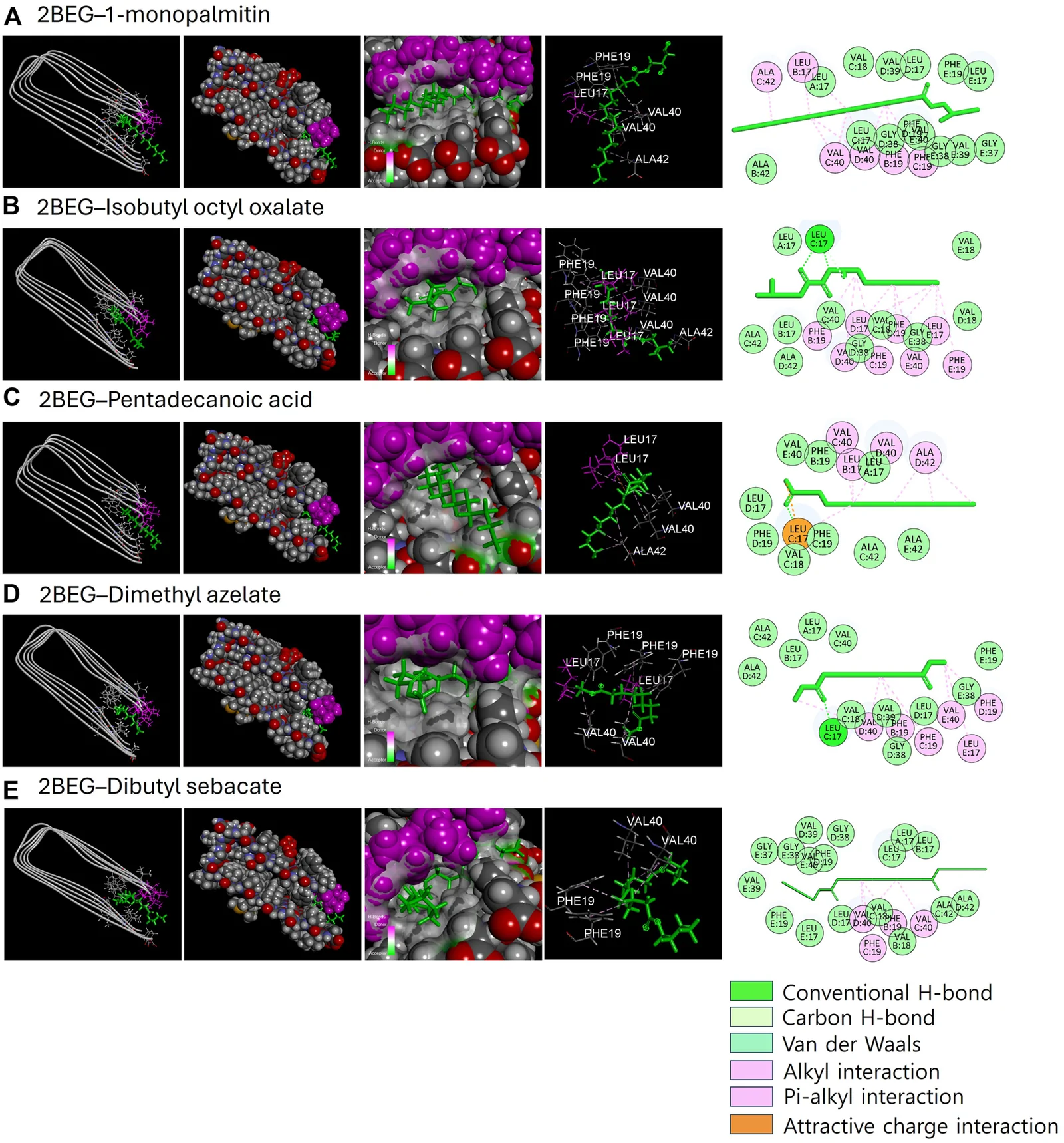
Molecular docking analysis of five JWS-derived compounds, including **(A)** 1-monopalmitin, **(B)** isobutyl octyl oxalate, **(C)** pentadecanoic acid, **(D)** dimethyl azelate, and **(E)** dibutyl sebacate, against the pentameric Aβ_42_ structure, PDB ID 2BEG. First panel shows the ligand positioned within the pentameric structure of Aβ, with interacting residues highlighted in gray and pink. Second panel shows a detailed atomic representation of Aβ in a ball-and-stick model, with the ligand shown in green and carbon, oxygen, and nitrogen atoms shown as gray, red, and blue spheres, respectively. Third panel shows an enlarged surface view of the ligand-binding region within pentameric Aβ, with hydrogen-bond donor and acceptor surfaces indicated in purple and green, respectively. Fourth panel shows a simplified two-dimensional interaction diagram of ligand-binding residues in Aβ. The labels show the amino acid names in three-letter code and their positions in the Aβ peptide. The fifth panel shows a two-dimensional interaction map of ligand-binding residues in Aβ. Circles indicate interacting residues, and colors indicate interaction types defined in the bottom panel.

Overall, the selected compounds showed stronger binding to the pentameric Aβ structure than to the monomeric forms, with hydrophobic interactions predominantly observed at aggregation-associated residues. Hydrophobic interactions were predominantly observed in the pentameric structure, whereas hydrogen bonding was more frequently observed in the monomeric structures.

### Anti-aggregation and dissociation effects of four JWS-derived compounds on Aβ_42_

To validate the inhibitory effects of the five selected JWS-derived compounds on Aβ_42_ aggregation predicted by *in silico* screening, ThT fluorescence assays were performed. Aβ_42_ was incubated in the presence or absence of 1-monopalmitin, isobutyl octyl oxalate, pentadecanoic acid, dimethyl azelate, and dibutyl sebacate at concentrations of 2.5, 25, and 250 μM, with morin at 100 μM as a positive control (Figure 5A). Among the five selected JWS-derived compounds, four compounds—1-monopalmitin, isobutyl octyl oxalate, pentadecanoic acid, and dimethyl azelate—inhibited Aβ_42_ aggregation, with 1-monopalmitin, isobutyl octyl oxalate, and dimethyl azelate showing concentration-dependent inhibition and pentadecanoic acid exhibiting significant inhibition only at 25 μM; dibutyl sebacate showed no significant inhibitory effect at any concentration tested. Morin reduced ThT fluorescence to 51.61% of the vehicle. Among the four selected compounds, 1-monopalmitin exhibited the strongest inhibitory activity against Aβ_42_ aggregation, reducing ThT fluorescence to 67.08%, 69.63%, and 86.58% at 250, 25, and 2.5 μM, respectively. Treatment with isobutyl octyl oxalate reduced ThT fluorescence to 73.03%, 81.81%, and 88.40% at 250, 25, and 2.5 μM, respectively. Meanwhile, pentadecanoic acid significantly inhibited Aβ_42_ aggregation only at 25 μM, reducing ThT fluorescence to 85.63%. Dimethyl azelate significantly reduced ThT fluorescence to 80.68% and 85.40% at 250 and 25 μM, respectively, without a significant effect at 2.5 μM. In addition, the crude JWS extract exhibited more potent inhibitory effects on Aβ_42_ aggregation than any individual compound, reducing ThT fluorescence to 40.14% and 33.07% at 400 and 600 μg/mL, respectively. These results suggest that multiple constituents within the JWS extract may collectively contribute to its stronger inhibitory activity against Aβ_42_ aggregation.

**FIGURE 5:**
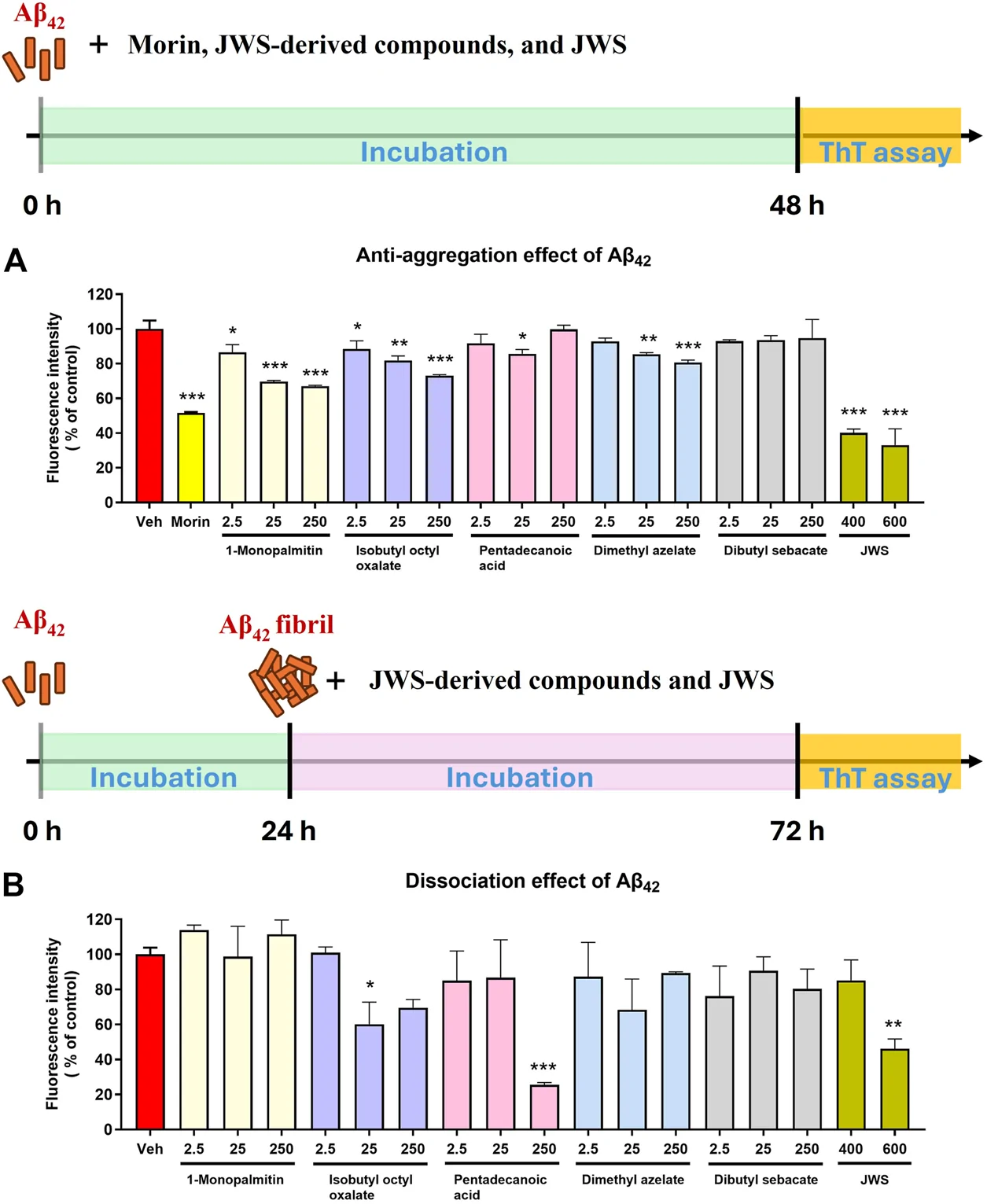
Effects of JWS-derived compounds on Aβ_42_ aggregation and dissociation *in vitro*. **(A)** Endpoint fluorescence values for the anti-aggregation effect were quantified and normalized to the vehicle-treated group. Aβ_42_ at 25 μM was incubated with 1-monopalmitin, isobutyl octyl oxalate, pentadecanoic acid, dimethyl azelate, and dibutyl sebacate at concentrations of 2.5, 25, or 250 μM for 48 h. Morin at 100 μM was used as a positive control for Aβ_42_ aggregation inhibition. The vehicle group was treated with the same volume of triple-distilled water. **(B)** Endpoint fluorescence values for the dissociation effect were quantified and normalized to the vehicle-treated group. Aβ_42_ was pre-aggregated for 24 h and then incubated with each compound at the same concentrations as in **(A)** for an additional 48 h. Data represent mean ± SEM of technical triplicates from a single experiment. Statistical analysis was performed using one-way ANOVA followed by Fisher’s LSD *post hoc* test. ^***^ *p* < 0.001, ^**^ *p* < 0.01, ^*^ *p* < 0.05 versus vehicle. Veh, Vehicle.

To further examine whether the selected compounds could dissociate preformed Aβ_42_ aggregates, pre-aggregated Aβ_42_ was separately incubated with each compound at 2.5, 25, and 250 μM (Figure 5B). Among the five compounds tested, only isobutyl octyl oxalate and pentadecanoic acid significantly dissociated preformed Aβ_42_ aggregates: isobutyl octyl oxalate reduced ThT fluorescence to 60.10% of the vehicle at 25 μM, while pentadecanoic acid showed a stronger dissociation effect at 250 μM, reducing ThT fluorescence to 25.59%. The crude JWS extract also reduced ThT fluorescence of preformed Aβ_42_ aggregates to 46.16% at 600 μg/mL. In contrast, 1-monopalmitin, dimethyl azelate, and dibutyl sebacate did not significantly dissociate preformed Aβ_42_ aggregates at any tested concentration. Collectively, these results identify isobutyl octyl oxalate and pentadecanoic acid as JWS-derived compounds with Aβ-disaggregating activity, with pentadecanoic acid exhibiting particularly potent dissociation activity that exceeded that of the JWS extract at the highest concentration tested.

### Molecular dynamics simulations of four JWS-derived compounds with Aβ

To examine the short-timescale interactions of the four selected compounds with Aβ, we performed 10,000-ps MD simulations of the Aβ_17-42_ dimer (PDB ID: 2BEG) in triplicate. The simulations were performed on the ligand-free Aβ_17-42_ dimer as the control and in complex with 1-monopalmitin, isobutyl octyl oxalate, pentadecanoic acid, and dimethyl azelate. Mean total energies from three independent simulations over 1,000–10,000 ps are summarized in Figure 6A, and the time-resolved triplicate total-energy data are provided in Supplementary Table S5. Time-resolved energy profiles are shown in Figures 6B–F, Supplementary Figures S4A–E, S5A–E. Representative structures at 50–10,000 ps are shown in Figures 6G–K, Supplementary Figures S4F–J, S5F–J. Across all five systems, total-energy profiles fluctuated around relatively stationary mean values without substantial progressive drift during the analyzed interval. Because the ligand-free and ligand-bound systems differed in molecular composition and total atom number, absolute mean total energies were not compared across systems and were not interpreted as measures of ligand-binding affinity or Aβ_17–42_ dimer stability. Accordingly, total-energy analysis was used only as a descriptive indicator of energetic stationarity and replicate reproducibility within each system. Accordingly, differences in mean total energy among the systems were not used to rank ligand–Aβ interactions or to validate the molecular docking results. Instead, the MD trajectories were examined qualitatively to assess short-timescale ligand behavior and ligand-associated structural changes at the Aβ_17–42_ dimer interface.

**FIGURE 6:**
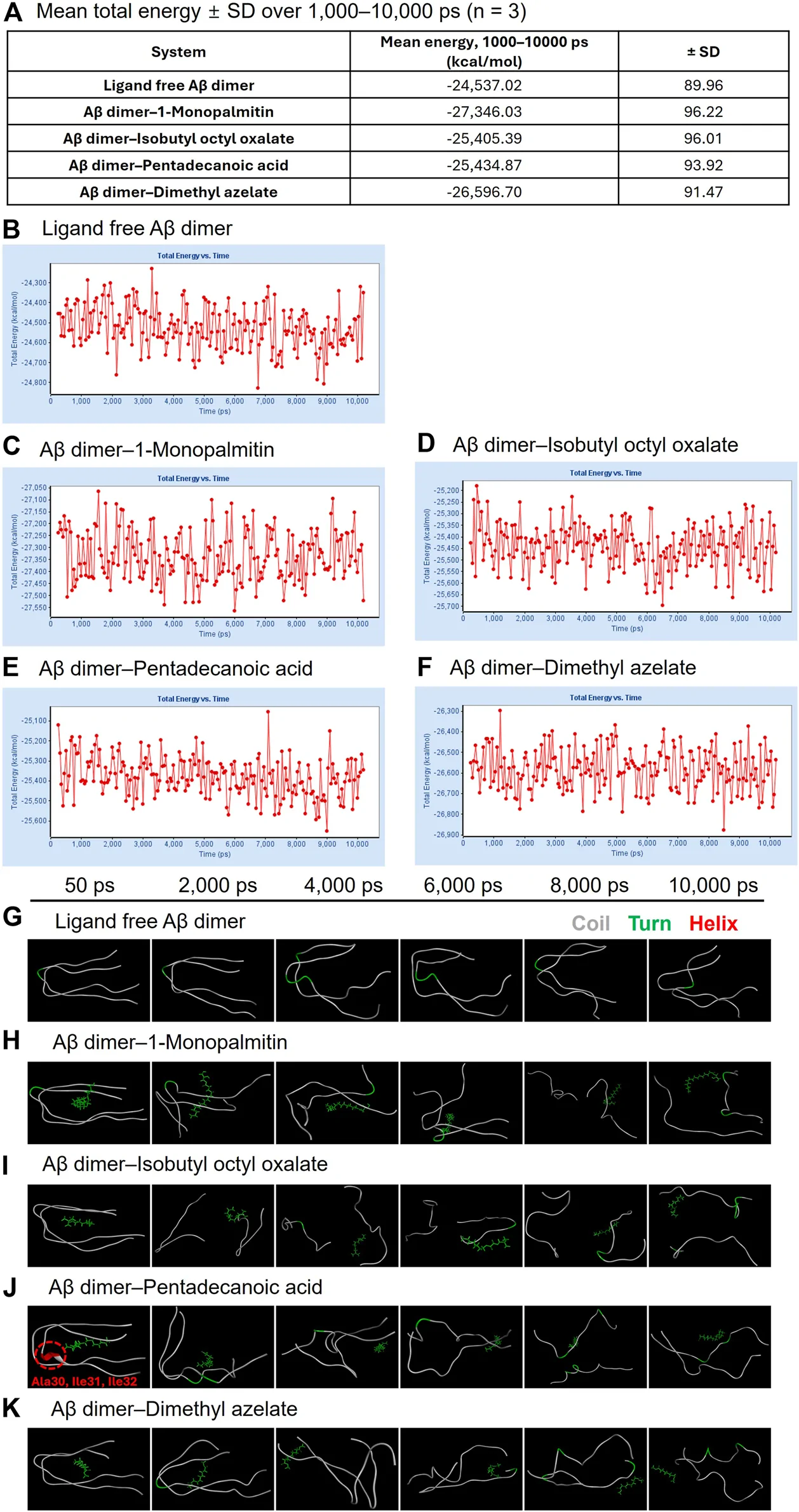
MD simulations of Aβ_17-42_ dimer-candidate compound complexes. **(A)** Mean total energy ±SD over 1,000–10,000 ps from three independent simulations, shown as a descriptive indicator of energetic stationarity and replicate reproducibility within each system. Absolute mean total-energy values were not interpreted as measures of ligand-binding affinity or Aβ dimer stability across systems. Total energy profiles of Aβ_17-42_ dimer-candidate compound complexes **(B–F)**. **(B)** Ligand-free Aβ_17-42_ dimer. **(C)** Aβ_17-42_ dimer complexed with 1-monopalmitin. **(D)** Aβ_17-42_ dimer complexed with isobutyl octyl oxalate. **(E)** Aβ_17-42_ dimer complexed with pentadecanoic acid. **(F)** Aβ_17-42_ dimer complexed with dimethyl azelate. The data are presented as total energy in kcal/mol versus time during production step throughout the simulation trajectory. MD simulations were performed for 10,000 ps to evaluate the structural alteration of the Aβ_17-42_ dimer both in the absence and in the presence of candidate compounds, with representative structural snapshots captured at several given time points throughout the simulation **(G–K)**. **(G)** Ligand-free Aβ_17-42_ dimer. **(H)** Aβ_17-42_ dimer complexed with 1-monopalmitin. **(I)** Aβ_17-42_ dimer complexed with isobutyl octyl oxalate. **(J)** Aβ_17-42_ dimer complexed with pentadecanoic acid. **(K)** Aβ_17-42_ dimer complexed with dimethyl azelate. Aβ peptide chains are shown as gray ribbons, with green segments indicating regions adopting turn conformations during the simulation. The four candidate compounds are represented as green sticks. Red dotted circles highlight residues that formed α-helical structures, and residue names are labeled adjacent to each circle.

MD simulations were performed to investigate the conformational dynamics of the dimeric Aβ peptide (PDB ID: 2BEG) over a 10,000 ps simulation in the presence and absence of four small-molecule compounds derived from JWS (Figure 6G). Representative structural snapshots of Aβ_17-42_ dimer were captured at 50 ps, 2,000 ps, 4,000 ps, 6,000 ps, 8,000 ps, and 10,000 ps to illustrate time-dependent structural evolution. In the ligand-free Aβ_17-42_ dimer, the two peptide chains remained closely associated throughout the representative snapshots, although conformational rearrangements were observed during the simulation (Figure 6G). The Aβ_17-42_ dimer—1-monopalmitin complex showed progressive separation at the dimer interface throughout the simulation, during which 1-monopalmitin frequently changed its position and orientation, with clear interchain separation observed after 8,000 ps (Figure 6H). The Aβ_17-42_ dimer complexed with isobutyl octyl oxalate displayed more pronounced structural rearrangements, including ligand-induced bending or twisting of chains, expansion of coil/turn segments, dissociation of Aβ dimers from 2,000 ps onward, and straightening of both Aβ peptides, particularly at 10,000 ps (Figure 6I). Similarly, the Aβ_17-42_ dimer complexed with pentadecanoic acid showed conformational rearrangements, including α-helix formation in residues Ala30, Ile31, and Ile32 at 2,000 ps time point and partial separation of the two Aβ peptides starting at 6,000 ps onward (Figure 6J). Finally, the Aβ_17-42_ complex with dimethyl azelate exhibited structural rearrangements, including the opening of the Aβ dimers at 4,000 ps, followed by partial separations between the two peptide chains at 6,000 ps and 10,000 ps (Figure 6K). Collectively, the ligand-containing trajectories showed varying degrees of dimer-interface rearrangement, partial chain separation, and changes in ligand position and orientation. These observations suggest transient ligand–Aβ interactions associated with early perturbation of the Aβ_17–42_ dimer interface rather than persistent stabilization of ligand-bound complexes. Given the limited 10,000-ps simulation period, these observations should be interpreted as short-timescale trajectory behavior and not as evidence of equilibrium binding or a complete fibril-disassembly pathway.

### RMSD analysis of four JWS-derived compounds with Aβ

To distinguish intra-chain conformational change from relative motion of the two peptide chains, backbone RMSD was recalculated after least-squares superposition onto the first production frame of each replica (Figure 7). Whole-dimer RMSD and chain-specific RMSD were obtained separately for the ligand-free Aβ_17–42_ dimer and for complexes with 1-monopalmitin, isobutyl octyl oxalate, pentadecanoic acid, and dimethyl azelate. In all five systems, fitted RMSD increased from 0 Å during the first ∼1,000 ps and then fluctuated without returning to the starting 2BEG geometry (Figures 7B–D). Mean backbone RMSD over the 1,000–10,000 ps window is summarized in Figure 7A. The ligand-free dimer yielded a mean dimer RMSD of 11.91 ± 2.16 Å, with chain A and chain B values of 10.24 ± 1.95 Å and 8.32 ± 2.29 Å, respectively. Mean dimer RMSD was 9.87 ± 2.05 Å for 1-monopalmitin, 12.39 ± 1.87 Å for isobutyl octyl oxalate, 9.81 ± 1.73 Å for pentadecanoic acid, and 8.98 ± 1.45 Å for dimethyl azelate. Per-chain means remained in a similar range and were generally comparable to or lower than the corresponding dimer values, except for dimethyl azelate, in which one replica produced higher chain-specific RMSD and therefore larger SDs for chains A and B (10.16 ± 4.82 Å and 10.60 ± 3.50 Å). Differences among the four ligand-bound complexes and the ligand-free dimer were smaller than replica-to-replica variation. Accordingly, the RMSD profiles were not interpreted as compound-specific stabilization of a 2BEG-like dimer, nor as evidence that the ligands remain in a single docked pose throughout the trajectory. Instead, the fitted RMSD data indicate that the Aβ_17–42_ dimer undergoes conformational rearrangement on the 10,000 ps timescale in both the absence and the presence of the candidate compounds. Mean dimer RMSD values for the four complexes (8.98–12.39 Å) were similar to that of the ligand-free dimer (11.91 ± 2.16 Å) and did not indicate a larger deviation from the starting geometry in the ligand-bound systems. Representative snapshots showed interface rearrangement and partial chain separation in some ligand-bound trajectories (Figures 6H–K); these visual changes were not mirrored by a systematic increase in fitted RMSD relative to the ligand-free dimer control (Figure 7).

**FIGURE 7:**
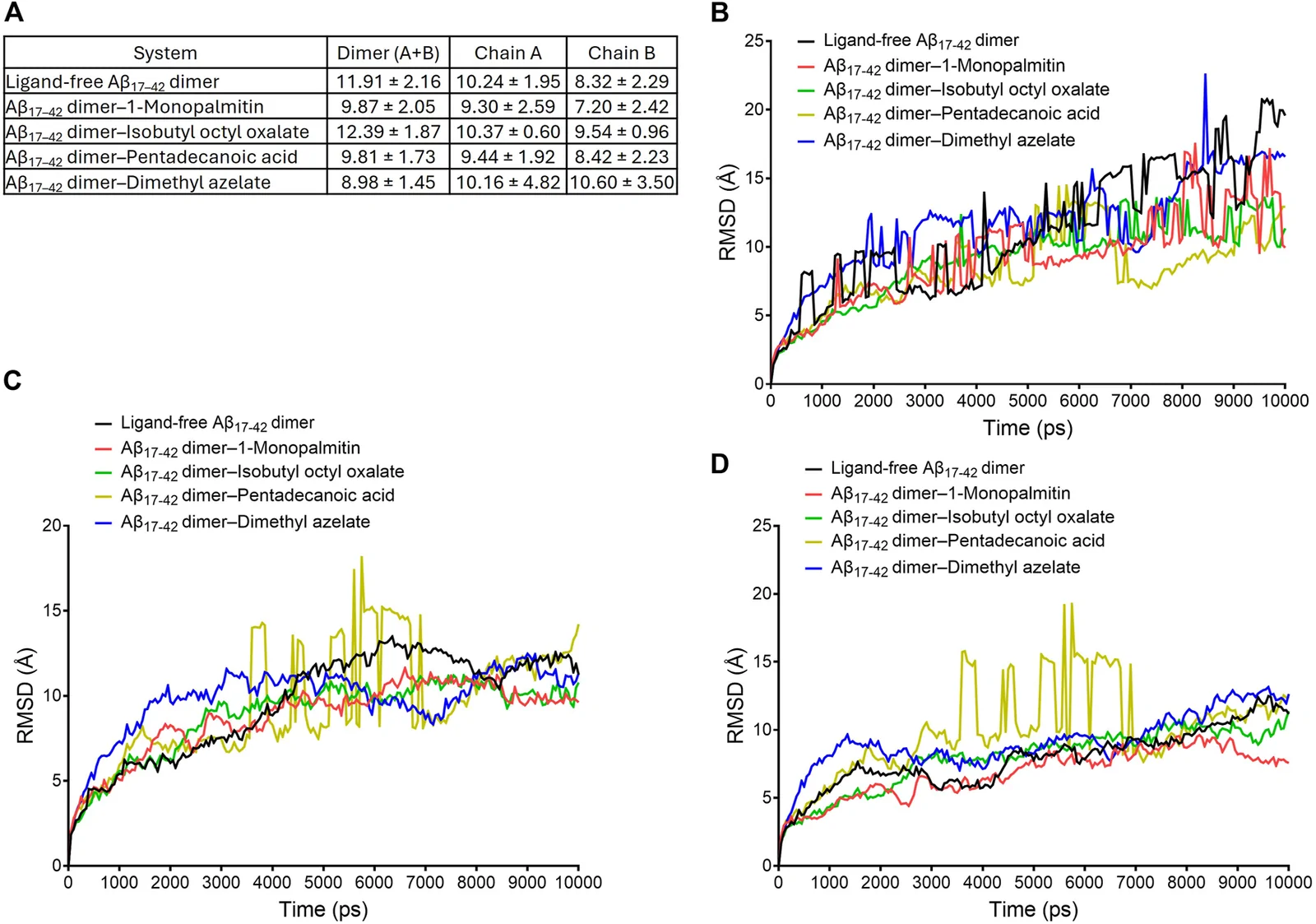
Backbone RMSD of the Aβ_17–42_ dimer after least-squares superposition onto the first production frame. **(A)** Mean ± SD (Å) over 1,000–10,000 ps from three independent replicas. **(B)** Dimer (chains A and B) versus time. **(C)** Chain A versus time. **(D)** Chain B versus time. Traces in **(B–D)** are means of three replicas and are shown for 0–10,000 ps.

### *In silico* prediction of pharmacokinetic properties and BBB permeability

ADME analysis plays a critical role in drug discovery and development for AD, as early-stage evaluation of pharmacokinetic properties is essential to reduce the risk of late-stage failure, particularly for compounds requiring effective brain exposure [37]. In this regard, *in silico* tools, such as PreADMET and pkCSM-pharmacokinetics, provide a rapid and reliable alternative to experimental approaches [36, 38]. Thus, we performed detailed ADME evaluation for the four JWS-derived candidate compounds using PreADMET. Additionally, their BBB permeability was verified using pkCSM-pharmacokinetics. To predict the pharmacokinetic properties for 1-monopalmitin, isobutyl octyl oxalate, pentadecanoic acid, and dimethyl azelate, we assessed key parameters including logP_o/w_, PPB, Caco-2 permeability, HIA, inhibition of CYP2C19 and CYP2D6 enzymes, total clearance, and BBB permeability (Table 4). The logP_o/w_ value was 4.36 for 1-monopalmitin, 3.08 for isobutyl octyl oxalate, 5.16 for pentadecanoic acid, and 2.06 for dimethyl azelate, all of which indicate lipophilic characteristics favorable for membrane permeability. In addition, all compounds exhibited a PPB value of 100%, suggesting strong plasma protein binding. A PPB value of 100% indicates reduced free drug fraction in circulation, which may influence BBB permeability and pharmacological efficacy [39]. In terms of intestinal absorption-related properties, the predicted values of Caco-2 permeability were 29.86 nm/s for 1-monopalmitin, 40.92 nm/s for isobutyl octyl oxalate, 25.04 nm/s for pentadecanoic acid, and 21.08 nm/s for dimethyl azelate. The HIA values were 91.41% for 1-monopalmitin, 96.06% for isobutyl octyl oxalate, 98.11% for pentadecanoic acid, and 93.42% for dimethyl azelate, indicating efficient intestinal absorption and suitability for oral administration. Regarding metabolic interactions, 1-monopalmitin, isobutyl octyl oxalate, pentadecanoic acid, and dimethyl azelate were predicted to inhibit CYP2C19, the whereas four JWS-derived candidate compounds were not predicted to inhibit CYP2D6. The predicted inhibition of CYP2C19 may indicate a potential for drug–drug interactions, which warrants further experimental validation. The predicted total clearance was 1.787 log mL/min/kg for 1-monopalmitin, 1.463 log mL/min/kg for isobutyl octyl oxalate, 1.528 log mL/min/kg for pentadecanoic acid, and 1.464 log mL/min/kg for dimethyl azelate. Moreover, the values of BBB penetration were 6.48 for 1-monopalmitin, 0.28 for isobutyl octyl oxalate, 6.64 for pentadecanoic acid, and 0.99 for dimethyl azelate. Taken together, these *in silico* predictions suggest that four JWS-derived compounds exhibit suitable absorption and metabolic profiles, whereas BBB permeability differs among them, with higher values observed for 1-monopalmitin and pentadecanoic acid compared with isobutyl octyl oxalate and dimethyl azelate.

**TABLE 4:** Pharmacokinetic properties of four JWS-derived compounds.

Compound	^a^LogP_o/w_	^b^PPB	^c^Caco-2	^d^HIA	^e^CYP2C19	^f^CYP2D6	^g^Total clearance	^h^BBB
1-Monopalmitin	4.36	100	29.86	91.41	Inhibitor	Non	1.787	6.48
Isobutyl octyl oxalate	3.08	100	40.92	96.06	Inhibitor	Non	1.463	0.28
Pentadecanoic acid	5.16	100	25.04	98.11	Inhibitor	Non	1.528	6.64
Dimethyl azelate	2.06	100	21.08	93.42	Inhibitor	Non	1.464	0.99

^a^ Values > 1 indicate lipophilicity; values <1 indicate hydrophilicity.

^b^ Values > 90% indicate strong plasma protein binding; values <90% indicate weak plasma protein binding.

^c^ Values < 20 nm/s indicate low intestinal epithelial permeability; 20–70 nm/s indicate moderate intestinal epithelial permeability; >70 nm/s indicate high intestinal epithelial permeability.

^d^ Values ≤ 20% indicate poor intestinal absorption; 20–70% moderate intestinal absorption; >70% high intestinal absorption.

^e^ Inhibitor indicates predicted inhibition of CYP2C19; Non indicates no inhibition.

^f^ Inhibitor indicates predicted inhibition of CYP2D6; Non indicates no inhibition.

^g^ Values are expressed as log_10_ mL/min/kg; higher values indicate increased clearance.

^h^ Values < 0.1 indicate low BBB, penetration; 0.1–2.0 indicate moderate BBB, penetration; >2 indicate high BBB penetration.

## Discussion

This study aimed to identify bioactive compounds of JWS involved in the regulation of Aβ pathology. Using an integrated *in silico* and *in vitro* workflow, we systematically identified small molecules from JWS with the potential to modulate aggregation and dissociation of Aβ (Figure 1). First, a curated library of 1,751 compounds derived from the 14 constituent herbs of JWS was established (Supplementary Table S1). Next, to identify compounds with potential brain-targeting properties, we screened the curated library using molecular weight, Lipinski’s rule-of-five, and predicted BBB permeability criteria. This process resulted in the selection of 566 compounds for subsequent screening (Supplementary Table S2). Additionally, multi-target docking simulations were performed using the 566 selected compounds against three representative Aβ structures, including α-helical Aβ monomer 1Z0Q, β-sheet Aβ monomer 2NAO, and Aβ pentameric protofibril 2BEG (Supplementary Table S3). Based on low CDOCKER energy values, the top 35 compounds were selected separately for each Aβ structural model. After excluding compounds that were not consistently identified across all three Aβ structural models, 13 distinct compounds remained. These 13 compounds were further filtered based on their specific docking interactions with aggregation-prone Aβ residues (Supplementary Table S4). Consequently, this process narrowed the selection down to five prioritized candidate compounds: 1-monopalmitin, isobutyl octyl oxalate, pentadecanoic acid, dimethyl azelate, and dibutyl sebacate. Notably, molecular docking simulations suggested that all five compounds had predicted Aβ-binding potential and formed interactions with aggregation-prone Aβ residues across the three Aβ structural models (Table 3; Figures 2–4). Subsequently, ThT fluorescence assays were carried out to evaluate the effects of five selected compounds on Aβ_42_ fibrillization and dissociation of mature Aβ_42_ fibrils in a cell-free system. ThT fluorescence assays showed that 1-monopalmitin, isobutyl octyl oxalate, pentadecanoic acid, and dimethyl azelate significantly inhibited Aβ_42_ aggregation, whereas isobutyl octyl oxalate and pentadecanoic acid also promoted dissociation of preformed Aβ_42_ aggregates (Figure 5). To further investigate the structural mechanisms underlying the inhibition of Aβ_42_ fibrillization and the dissociation of mature Aβ_42_ fibrils observed in the ThT assay, MD simulations were performed using the four compounds: 1-monopalmitin, isobutyl octyl oxalate, pentadecanoic acid, and dimethyl azelate. Dibutyl sebacate was not included in MD simulations, since it showed no significant effect on either Aβ_42_ fibrillization or mature Aβ_42_ fibril dissociation. In MD simulations, representative snapshots of the ligand-containing systems showed rearrangement at the Aβ_17–42_ dimer interface that was more apparent than in the selected ligand-free frames (Figure 6). Fitted backbone RMSD increased during the first ∼1,000 ps and then fluctuated around system-specific values (Figure 7). Mean dimer RMSD over 1,000–10,000 ps was similar for the ligand-free dimer and the four complexes and was not interpreted as ligand-specific stabilization or as an absence of conformational change (Figure 7). Finally, *in silico* pharmacokinetic prediction suggested that the four JWS-derived compounds exhibit favorable pharmacological profiles, whereas 1-monopalmitin and pentadecanoic acid showed higher predicted BBB permeability than isobutyl octyl oxalate and dimethyl azelate (Table 4). These results support the effectiveness of combining *in silico* and *in vitro* methods to discover JWS-derived candidates as Aβ inhibitors and to identify the JWS-derived candidate molecules for controlling Aβ pathology.

To understand the biological relevance of the identified molecules, we examined their botanical origins based on the compound informatics (TM-MC 2.0) used for library construction [34]. According to the database, 1-monopalmitin and dimethyl azelate are listed as constituents of *Zizyphi Semen* and *Schisandrae Fructus*, respectively [40–44]. Isobutyl octyl oxalate is identified as a component of *Platycodi Radix* [45, 46]. Although specific isolation methods for alkyl esters, such as dimethyl azelate, are limited in the literature, their presence in the herbal database suggests they may represent bioactive lipid fractions of the parent herbs. Importantly, these herbs—*Zizyphi Semen*, *Schisandrae Fructus*, and *Platycodi Radix*—have shown significant modulatory effects on Aβ pathology in preclinical studies, including antioxidant activity and inhibition of Aβ production and deposition, leading to a reduced amyloid burden [40–46]. Furthermore, pentadecanoic acid, an odd-chain saturated fatty acid with emerging neuroprotective properties, has been detected in lipid profiles of *Coicis Semen*, an ingredient of JWS [47]. Although the general neuroprotective properties of *Coicis Semen* are known [47, 48], its direct role in inhibiting Aβ aggregation or reducing amyloid burden remains unclear.

Virtual screening has emerged as a powerful strategy in drug discovery, particularly for neurological disorders, by enabling efficient *in silico* evaluation of vast compound libraries and thereby reducing reliance on animal testing [35, 49]. The integration of *in silico* screening with complementary *in vitro* validation, as demonstrated here for Aβ inhibitors derived from JWS, improves the efficiency of hit compound identification, accelerates lead optimization, and reduces the time and financial costs associated with traditional preclinical development [50–52]. By prioritizing potential candidates through computational filtering, virtual screening facilitates broader exploration of chemical space, identifies novel compounds with high biological activity as starting points for optimization, and supports the replace, reduce, refine (3Rs) principle in animal use [49]. Consequently, the pharmaceutical industry is increasingly adopting computational platforms to optimize drug discovery pipelines while maintaining rigorous validation [49, 53].

Molecular docking is a useful *in silico* approach for predicting compound-target interactions and refining potential therapeutic candidates in modern drug discovery [54]. In the present study the four JWS-derived compounds preferentially interacted with aggregation-prone hydrophobic regions of Aβ_42_, particularly the central ^16^KLVFFA^21^ region and the hydrophobic C-terminal region [23]. Interaction-frequency analysis showed that residues 15-21 represented the predominant interaction region for all four compounds, whereas residues 36-42 were the second most frequently involved region (Supplementary Figure S3). This binding pattern is consistent with previous research showing that natural compounds can inhibit Aβ aggregation by directly targeting hydrophobic residues involved in oligomer nucleation and fibril elongation [35, 55–57]. For example, morin and datiscetin interact with the ^19^FFA^21^ region as well as the ^10^YEVHHQ^15^ sequence of Aβ_42_ monomers and inhibit both the nucleation and elongation phases of amyloid aggregation, thereby reducing Aβ-induced neuronal toxicity [58]. Quercetin recognizes the ^17^LVFFA^21^ region of Aβ oligomers during the early stages of aggregation, suggesting that interaction with this central hydrophobic region may interfere with early Aβ assembly [59]. Similarly, baicalein binds several aggregation-prone residues within both central and C-terminal regions, such as Leu17, Val18, Phe19, Ala21, Val24, Val36, Val40, and Ala42, and disrupts β-sheet formation and protofibril stability [35]. Natural cembranoid and tanshinone derivatives have been reported to preferentially interact with hydrophobic residues within the C-terminal region of Aβ_42_, supporting the importance of this region in modulating aggregate formation and stability [60, 61]. In addition, epigallocatechin gallate (EGCG) binds preferentially to Aβ oligomers and fibrils through interactions with residues, such as Phe19, Lys28, Ala30, and Ile31, while disrupting N-terminal salt bridges to remodel aggregates into non-toxic off-pathway species [62, 63]. Moreover, resveratrol has been reported to interact with hydrophobic Aβ residues, including Val12, His13, Lys16, Phe20, Asp23, Val24, and Ile31, and this interaction may promote cleavage at Arg5-His6 and Val24-Gly25, thereby fragmenting Aβ peptide and interfering with peptide aggregation [55, 56]. Therefore, these previous studies demonstrate that natural Aβ aggregation inhibitors can act at different stages of aggregation by targeting distinct regions of the peptide. In this regard, the four JWS-derived compounds showed binding profiles similar to those of natural inhibitors targeting the central hydrophobic region, and their substantial interactions with residues 36-42 resembled those of compounds that engage the hydrophobic C-terminal region. Since these regions contribute to Aβ self-assembly, fibril elongation, and fibril stability, preferential binding of the JWS-derived compounds to these sites may interfere with multiple processes of Aβ aggregation. In contrast, some inhibitors, such as nicotine, preferentially interact with N-terminal histidine residues [64], indicating that natural compounds can modulate Aβ aggregation through different residue-specific mechanisms. The functional relevance of this binding pattern is supported by the ThT results, in which all four compounds significantly inhibited Aβ_42_ aggregation/fibrilization (Figure 5). However, the magnitude of inhibition differed among the compounds and concentrations. Among the four compounds, 1-monopalmitin showed a relatively strong inhibitory effect on Aβ_42_ aggregation, whereas isobutyl octyl oxalate and dimethyl azelate exhibited greater inhibition at higher concentrations. By contrast, pentadecanoic acid did not show a clear dose-dependent effect in the ThT assay but, like isobutyl octyl oxalate, promoted the dissociation of preformed Aβ_42_ fibrils. These different patterns suggest that the identified compounds may differ in their ability to modulate Aβ fibril formation and the stability of preformed fibrils.

Since molecular docking provides static binding poses, MD simulations were further performed to examine the short-timescale behavior of the predicted compound–Aβ interactions and any ligand-associated structural changes in the Aβ_17–42_ dimer. MD simulations enable atomistic evaluation of ligand-protein interactions over time and provide insight into conformational changes that cannot be fully captured by molecular docking alone [65]. This dynamic evaluation is particularly relevant for Aβ, since its aggregation is closely associated with conformational rearrangements that promote β-sheet-rich assemblies and fibril formation [35, 66]. In addition, previous studies have shown that transient interactions between small molecules and Aβ peptides can effectively modulate the nucleation process and Aβ assemblies [67, 68]. In the present study, the four compounds—1-monopalmitin, isobutyl octyl oxalate, pentadecanoic acid, and dimethyl azelate—formed both initial and subsequent transient contacts with the Aβ_17-42_ dimer during the 10,000 ps simulations. Fitted backbone RMSD showed conformational deviation from the first production frame in the ligand-free and ligand-bound systems, without a systematic ligand-dependent increase relative to the ligand-free dimer (Figure 7). Notably, representative snapshots showed ligand-associated rearrangement of the Aβ_17–42_ dimer, ranging from interface opening with residual chain contact (Figures 6J,K) to extensive inter-chain separation (Figures 6H,I). These visual changes suggest that the compounds can perturb the starting dimeric packing on the simulation timescale, but they were not interpreted as complete or irreversible dimer dissociation, and they were not accompanied by a systematic increase in fitted RMSD relative to the ligand-free control (Figure 7).

Although the multi-step screening approach provides valuable mechanistic insights into compound-Aβ interactions, several limitations should be acknowledged. Molecular docking provides predicted binding poses and relative scoring estimates rather than direct measurements of binding affinity or biological activity. Therefore, the docking scores and predicted interactions identified in this study should be interpreted as hypothesis-generating computational evidence, and the predicted compound–Aβ interactions require further experimental validation using appropriate biophysical binding assays. First, the computational analyses relied on a restricted set of Aβ structures, which may not fully represent the conformational heterogeneity of Aβ monomers, oligomers, and fibrillar assemblies [69, 70]. In addition, docking scores (CDOCKER energy) are empirical pose-ranking metrics and should not be interpreted as experimental binding affinities. Second, MD simulations were limited to 10,000 ps of production sampling of the Aβ_17–42_ dimer (2BEG). This timescale is sufficient to inspect short-term pose persistence and early ligand-induced changes at the dimer interface, but it is not sufficient to capture nucleation, higher-order oligomerization, rare unbinding events, or the full pathway of fibril disassembly. Although production runs were performed in triplicate with independent initial velocities and yielded consistent qualitative interface perturbation, three 10,000-ps replicas do not substitute for microsecond-scale or enhanced-sampling simulations. Third, we did not compute MM/PBSA (or related) binding free energies. Such end-point methods are most appropriate for relatively persistent complexes in well-defined pockets; they are less reliable when conformational entropy is large, the binding interface is poorly defined, or sampling is incomplete [71, 72]. They are therefore less suitable for shallow, transient contacts on an Aβ oligomer, which is the binding mode suggested by our trajectories and by previous studies of small-molecule–Aβ interactions [67, 68]. Accordingly, computational results are used only to generate a mechanistic hypothesis for how the four compounds may engage aggregation-prone hydrophobic residues. Experimental support for anti-aggregation and disaggregation activity is provided by ThT assays, not by docking energies, MD total-energy values, or global RMSD profiles. Another limitation is that the experimental validation was conducted in a cell-free system. Therefore, key biological aspects, including neuronal toxicity, oligomer-induced cytotoxicity, intracellular uptake, BBB penetration, pharmacokinetics, and *in vivo* efficacy, remain to be evaluated in appropriate AD animal models.

Furthermore, several translational considerations should be addressed before advancing these JWS-derived candidates toward further development. First, the therapeutic significance of targeting Aβ aggregation should be considered within the broader context of Aβ production and clearance. Cerebral Aβ accumulation reflects an imbalance between Aβ production and clearance, and impaired clearance may contribute to amyloid accumulation even in the absence of increased Aβ production [23]. Recent perspectives on AD pathogenesis have further highlighted dysfunction of physiological brain clearance mechanisms, including the glymphatic system, as a potential contributor to Aβ accumulation [73]. The glymphatic system supports the removal of interstitial solutes, including Aβ, through cerebrospinal fluid-interstitial fluid exchange, and its function is closely associated with sleep and vascular physiology [73, 74]. From this perspective, inhibition of Aβ aggregation may be most relevant as a complementary strategy that acts together with physiological clearance rather than as an isolated approach directed solely at protein aggregation. By limiting the formation and stability of aggregation-prone Aβ assemblies, the identified compounds may complement endogenous clearance processes rather than acting solely on protein aggregation. However, the present study did not evaluate Aβ clearance or glymphatic function, and whether the identified compounds influence these processes remains to be determined. Moreover, the translational feasibility of the identified compounds depends on their actual abundance in JWS and their ability to reach the brain at pharmacologically meaningful levels. An important limitation of the present study is that the concentrations of the four candidate compounds in the parent JWS extract were not quantitatively determined. Therefore, it remains unclear whether these compounds are present at levels sufficient to account for the previously reported anti-Aβ activity of JWS. Quantitative analysis of these compounds in the JWS extract using LC-MS or HPLC, together with comparison of their measured concentrations with effective concentrations identified in the present biochemical assays and assessment of their exposure in relevant biological matrices, will be necessary to clarify their contribution to the biological activity of JWS. Beyond their abundance in the parent extract, the translational relevance of these candidates depends on whether they can reach CNS targets at pharmacologically meaningful levels. Given that Aβ pathology develops within the AD brain, brain delivery is a critical determinant of therapeutic feasibility. Although four compounds—1-monopalmitin, isobutyl octyl oxalate, pentadecanoic acid, and dimethyl azelate—showed acceptable predicted BBB permeability, their predicted PPB values approached 100% (Table 4), indicating that the free unbound fraction available for tissue distribution and pharmacological activity may be substantially limited. Therefore, future optimization using physiologically based pharmacokinetic modeling, targeted formulations, or conjugate-based delivery strategies may be required to improve exposure, bioavailability, and translational feasibility.

## Conclusion

The main objective of this study is to identify JWS-derived candidate compounds with the potential to modulate Aβ aggregation and disaggregation. Here, we present an end-to-end workflow to identify JWS-derived compounds that modulate Aβ pathology. Our integrated approach combines SwissADME screening for drug-likeness and BBB permeability, multi-conformational docking against Aβ, ThT validation, MD simulations, and ADME profiling. We identified four JWS-derived compounds—1-monopalmitin, isobutyl octyl oxalate, pentadecanoic acid, and dimethyl azelate—that showed modulatory effects on Aβ aggregation and dissociation in cell-free biochemical assays. Molecular docking and MD simulations suggested that four selected compounds preferentially bind to hydrophobic aggregation-prone regions of Aβ, potentially disrupting key electrostatic interactions critical for fibril stability. Additionally, ThT assays validated the integrated *in silico* approach, with all four compounds significantly inhibiting Aβ_42_ aggregation, while isobutyl octyl oxalate and pentadecanoic acid exhibited disaggregative effects on preformed Aβ_42_ aggregates. Furthermore, pharmacokinetic profiling using SwissADME predicted that 1-monopalmitin, isobutyl octyl oxalate, pentadecanoic acid, and dimethyl azelate possess drug-like characteristics, including compliance with key physicochemical parameters and predicted BBB permeability. Taken together, these findings identify the JWS-derived compounds as preliminary candidates for further investigation of their Aβ-modulating activity. However, given that the present study is limited to computational analyses and cell-free biochemical assays, their therapeutic relevance in AD remains to be established. Further cellular, pharmacological, toxicological, and *in vivo* studies are required to validate their biological activity, brain exposure, safety, and therapeutic efficacy before their potential for further development can be determined.

## Data Availability

The original contributions presented in the study are included in the article/Supplementary Material, further inquiries can be directed to the corresponding authors.

## Footnotes

1
https://tm-mc.kr/search.do

2
https://pubchem.ncbi.nlm.nih.gov/

3
https://www.swissadme.ch

4
https://preadmet.webservice.bmdrc.org/

5
https://biosig.lab.uq.edu.au/pkcsm
