# Supplementary material for: Integrated computational screening and biochemical validation identify natural small molecules targeting Aβ aggregation and dissociation
Source: J Pharm Pharm Sci. 2026 Oct 20;29:17216. doi: 10.3389/jpps.2026.17216 (PMC13727513; doi:10.3389/jpps.2026.17216)
Supplement: Supplementary file 1 [file Supplementaryfile1.docx]

**Supplementary figures**

**Supplementary figure 1.** Chemical structures of the five selected compounds derived from JWS. Chemical structures were obtained from the PubChem database.

**Supplementary figure 2. Evaluation of the effect of DMSO on Aβ_42_ aggregation.** Aβ_42_ was incubated with distilled water or dimethyl sulfoxide (DMSO) at final concentrations of 0.019%, 0.19%, and 1.9% DMSO for 48 h. Aβ_42_ aggregation was subsequently assessed using the thioflavin T (ThT) assay. Fluorescence intensity was normalized to DW-treated control and expressed as a percentage of the control. Each condition was measured in two technical replicate wells within a single experiment, and data are presented as mean ± SEM. Statistical analysis was performed using one-way ANOVA followed by Fisher’s LSD post hoc test.

 **Supplementary figure 3. Interaction profiles of four JWS-derived compounds with Aβ_42_.** Interaction of 1-monopalmitin, isobutyl octyl oxalate, pentadecanoic acid, and dimethyl azelate with three different forms of Aβ_42_ were analyzed using Discovery Studio. Interacting amino acids residues were grouped according to their positions in the Aβ_42_ sequence as residues 1-7, 8-14, 15-21, 22-28, 29-35, and 36-42. The graphs show the percentage distribution of interactions within each residue region for each compound.

**Supplementary figure 4. MD simulations of Aβ_17-42_ dimer-candidate compound complexes of replicate 2.** Total energy profiles of Aβ_17-42_ dimer-candidate compound complexes (A–E). (A) Ligand‑free Aβ_17-42_ dimer. (B) Aβ_17-42_ dimer complexed with 1-monopalmitin. (C) Aβ_17-42_ dimer complexed with isobutyl octyl oxalate. (D) Aβ_17-42_ dimer complexed with pentadecanoic acid. (E) Aβ_17-42_ dimer complexed with dimethyl azelate. The data are presented as total energy in kcal/mol versus time during production step throughout the simulation trajectory. MD simulations were performed for 10,000 ps to evaluate the structural alteration of the Aβ_17-42_ dimer both in the absence and in the presence of candidate compounds, with representative structural snapshots captured at several given time points throughout the simulation (F–J). (F) Ligand-free Aβ_17-42_ dimer. (G) Aβ_17-42_ dimer complexed with 1-monopalmitin. (H) Aβ_17-42_ dimer complexed with isobutyl octyl oxalate. (I) Aβ_17-42_ dimer complexed with pentadecanoic acid. (J) Aβ_17-42_ dimer complexed with dimethyl azelate. Aβ peptide chains are shown as gray ribbons, with green segments indicating regions adopting turn conformations during the simulation. The four candidate compounds are represented as green sticks.

**Supplementary figure 5. MD simulations of Aβ_17-42_ dimer-candidate compound complexes of replicate 3.** Total energy profiles of Aβ_17-42_ dimer-candidate compound complexes (A–E). (A) Ligand‑free Aβ_17-42_ dimer. (B) Aβ_17-42_ dimer complexed with 1-monopalmitin. (C) Aβ_17-42_ dimer complexed with isobutyl octyl oxalate. (D) Aβ_17-42_ dimer complexed with pentadecanoic acid. (E) Aβ_17-42_ dimer complexed with dimethyl azelate. The data are presented as total energy in kcal/mol versus time during production step throughout the simulation trajectory. MD simulations were performed for 10,000 ps to evaluate the structural alteration of the Aβ_17-42_ dimer both in the absence and in the presence of candidate compounds, with representative structural snapshots captured at several given time points throughout the simulation (F–J). (F) Ligand-free Aβ_17-42_ dimer. (G) Aβ_17-42_ dimer complexed with 1-monopalmitin. (H) Aβ_17-42_ dimer complexed with isobutyl octyl oxalate. (I) Aβ_17-42_ dimer complexed with pentadecanoic acid. (J) Aβ_17-42_ dimer complexed with dimethyl azelate. Aβ peptide chains are shown as gray ribbons, with green segments indicating regions adopting turn conformations during the simulation. The four candidate compounds are represented as green sticks.
